# Inflammasomes as Potential Therapeutic Targets to Prevent Chronic Active Viral Myocarditis—Translating Basic Science into Clinical Practice

**DOI:** 10.3390/ijms262211003

**Published:** 2025-11-13

**Authors:** Natalia Przytuła, Jakub Podolec, Tadeusz Przewłocki, Piotr Podolec, Anna Kabłak-Ziembicka

**Affiliations:** 1 Department of Cardiac and Vascular Diseases, The St. John Paul II Hospital, Prądnicka 80, 31-202 Kraków, Poland; 2 Department of Interventional Cardiology, Institute of Cardiology, Jagiellonian University Medical College, św. Anny 12, 31-007 Kraków, Poland; 3 Department of Interventional Cardiology, The St. John Paul II Hospital, Prądnicka 80, 31-202 Kraków, Poland; 4 Department of Cardiac and Vascular Diseases, Institute of Cardiology, Jagiellonian University Medical College, św. Anny 12, 31-007 Kraków, Poland; 5 Noninvasive Cardiovascular Laboratory, The St. John Paul II Hospital, Prądnicka 80, 31-202 Kraków, Poland

**Keywords:** myocarditis, inflammasomes, microRNAs, Interleukins, viral, bacterial, chronic active, pharmacological strategies

## Abstract

Despite substantial progress in medical care, acute myocarditis remains a life-threatening disorder with a sudden onset, often unexpectedly complicating a simple and common upper respiratory tract infection. In most cases, myocarditis is triggered by viral infections (over 80%), with an estimated incidence of 10–106 per 100,000 annually. The clinical course may worsen in cases of mixed etiology, where a primary viral infection is complicated by secondary bacterial pathogens, leading to prolonged inflammation and an increased risk of progression to chronic active myocarditis or dilated cardiomyopathy. We present a case report illustrating the clinical problem of acute myocarditis progression into a chronic active form. A central element of host defense is the inflammasome—an intracellular complex that activates pyroptosis and cytokine release (IL-1β, IL-18). While these processes help combat pathogens, their persistent activation may sustain inflammation and trigger heart failure and cardiac fibrosis, eventually leading to dilated cardiomyopathy. In this review, we summarize the current understanding of inflammasome pathways and their dual clinical role in myocarditis: they are essential for controlling acute infection but may become harmful when overactivated, contributing to chronic myocardial injury. Additionally, we discuss both novel and established therapeutic strategies targeting inflammatory and anti-fibrotic mechanisms, including IL-1 receptor blockers (anakinra, canakinumab), NOD-like receptor protein 3 (NLRP3) inhibitors (colchicine, MCC950, dapansutrile, INF200), NF-κB inhibitors, and angiotensin receptor-neprilysin inhibitors (ARNI), as well as microRNAs. Our aim is to emphasize the clinical importance of early identification of patients at risk of transitioning from acute to chronic inflammation, elucidate the role of inflammasomes, and present emerging therapies that may improve outcomes by balancing effective pathogen clearance with limitation of chronic cardiac damage.

## 1. Introduction

### 1.1. Epidemiology of Myocarditis

Despite substantial progress in medical care, acute myocarditis remains a life-threatening disorder with a sudden onset, often unexpectedly complicating a simple and common upper respiratory tract infection [1,2,3]. The annual incidence of myocarditis is reported to range from 10.2 to 105.6 per 100,000 population, with approximately 1.8 million cases occurring worldwide each year [4]. The etiology in most cases is viral (over 85%) [5]. Bacterial myocarditis accounts for an estimated 3–6% of cases, though available data remain limited [6]. Sporadic cases result from parasitic (e.g., *Trichinella spiralis*, *Schistosoma* spp.) and protozoal infections (e.g., *Toxoplasma gondii*, *Trypanosoma cruzi*—the causative agent of Chagas’s disease), as well as from spirochetes such as *Borrelia burgdorferi*, responsible for Lyme disease [2,7]. Noninfectious myocarditis may arise from drug toxicity or autoimmune disorders [7,8].

Viral myocarditis (VMC) can be induced by various viruses. Coxsackievirus B3 (CVB3, an enterovirus) is the most common cause of myocardial injury globally (34.6 per 1000 cases), followed by Influenza B (17.4 per 1000 cases), Influenza A (11.7 per 1000 cases), Coxsackievirus A (CVA, 9.1 per 1000 cases), and Cytomegalovirus (CMV) (8.0 per 1000 cases) [2,9].

In recent years, cardiotropic viruses utilizing angiotensin-converting enzyme 2 (ACE2), such as severe acute respiratory syndrome coronavirus 2 (SARS-CoV-2, responsible for COVID-19), have also been implicated in acute myocarditis [7,10,11,12]. A pooled analysis by Zuin et al. involving more than 20 million individuals revealed that myocarditis was nearly 2.5 times more prevalent in post-COVID-19 patients compared to controls [13]. Myocarditis occurred in 0.21 per 1000 patients recovering from COVID-19 (95% CI: 0.13–0.42), compared to 0.09 per 1000 control subjects (95% CI: 0.07–0.12) [13]. These findings indicate that COVID-19 may predispose patients to cardiac complications following otherwise mild respiratory infections. Furthermore, patients recovered from COVID-19 demonstrated an increased risk of incident myocarditis within one year of infection (HR: 5.16, 95% CI: 3.87–6.89; *p* < 0.0001) [14]. Conversely, COVID-19 vaccination has also been associated with myocarditis, with an incidence ranging from 8.1 to 39 cases per 100,000 persons (or doses) [15].

Bacterial myocarditis occurs more frequently in immunocompromised individuals or as part of systemic infections. Among bacterial pathogens, Gram-positive organisms such as *Staphylococcus aureus*, *Streptococcus pyogenes*, and *Corynebacterium diphtheriae* are most often identified. Less commonly, Gram-negative bacteria such as Neisseria meningitidis, *Haemophilus influenzae*, *Salmonella* spp., *Klebsiella* spp., and *Borrelia burgdorferi* are involved. Pathogenesis may involve either direct myocardial invasion or immune-mediated injury. Although bacterial myocarditis is less frequent than viral forms, early diagnosis is crucial due to its severity and the need for pathogen-specific antimicrobial therapy [5,7,16].

*Streptococcus pneumoniae* is not typically described as a direct cause of myocarditis; however, it remains a leading cause of pneumonia—accounting for up to 15% of community-acquired pneumonia (CAP) cases in the United States and 27% globally [17]. Given the high incidence of pneumococcal pneumonia and frequent hospitalizations, especially among elderly individuals, invasive pneumococcal disease is often accompanied by cardiac complications, most notably new-onset or worsening heart failure (HF) and cardiac arrhythmias [18,19,20].

### 1.2. Diagnostic Work-Ups

Patients presenting to the cardiology department with suspected myocarditis often exhibit specific symptoms such as chest pain, palpitations, shortness of breath, syncope, malaise, general weakness, and fatigue. A thorough medical history is essential to identify potential causes and triggers, including recent viral infections, toxin exposure, medication use, and family history of inflammatory myocardial or pericardial syndromes, cardiomyopathy, or sudden cardiac death [21,22].

The initial diagnostic test upon admission to a cardiac care unit is typically a 12-lead electrocardiogram (ECG) [21]. ECG frequently reveals changes suggestive of myocardial ischemia, often mimicking acute coronary syndrome (ACS) [22]. Recently, Ramantauskaitė et al. demonstrated that such ischemic ECG changes are clinically relevant [23]. They found that left ventricular (LV) ejection fraction (EF) in patients presenting with ST-segment elevation (STE) myocardial infarction (MI) was significantly lower compared to those with a non-STE pattern. The STE pattern was also associated with higher C-reactive protein (CRP) and troponin levels, along with reduced LV strain and lower LV-EF on echocardiography [23].

Echocardiography is the first-line imaging modality in all suspected cases of myocarditis, serving to rule out alternative diagnoses and detect ventricular dysfunction, pericardial effusion, and complications such as intracardiac thrombus [24,25]. There is an increasing role for LV strain assessment, which provides important prognostic information regarding adverse outcomes in myocarditis [26]. Echocardiographic evaluation focuses on both LV and right ventricular (RV) systolic function, LV dilatation, and diastolic function. It also allows for the assessment of LV strain, which is fundamental for prognostication. This examination is highly valuable for predicting patient recovery or deterioration.

Cardiac magnetic resonance (CMR) is recommended as the first-line diagnostic tool for establishing the diagnosis of uncomplicated myocarditis [27,28]. When acute myocarditis is suspected, CMR should be performed urgently and include T1/T2 mapping to support a confirmatory diagnosis [28]. The diagnosis of myocarditis is based on the Lake Louise Criteria, which are applied during CMR to identify myocardial inflammation, edema, and fibrosis. It is strongly recommended to confirm the diagnosis in clinically stable patients when the initial work-up suggests myocarditis, with CMR performed on admission and repeated as necessary [22,24].

Cardiac biopsy is particularly useful in acute myocarditis for confirming the final diagnosis and predicting outcomes, although it carries an increased risk of pericardial hematoma. In routine clinical practice, the Dallas Criteria are commonly accepted histopathological criteria for establishing a precise diagnosis of myocarditis [22,24]. According to these criteria, active myocarditis is defined by the presence of an inflammatory infiltrate within the myocardium, predominantly lymphocytic, accompanied by myocyte necrosis not typical of ischemic injury. Borderline myocarditis is characterized by the presence of inflammatory infiltrates without clear evidence of myocyte necrosis. Despite limitations such as sampling error and interobserver variability, the Dallas Criteria provide essential confirmation of myocarditis in high-risk patients and guide further therapeutic decisions [22,29]. Endomyocardial biopsy remains necessary in high-risk cases to guide therapy and was recommended as class I by recent guidelines [24]. Both diagnostic tools provide insight into the extent of inflammation and fibrosis, which are crucial for predicting the future course of myocarditis [1,22,24,29,30,31].

**Figure 1:**
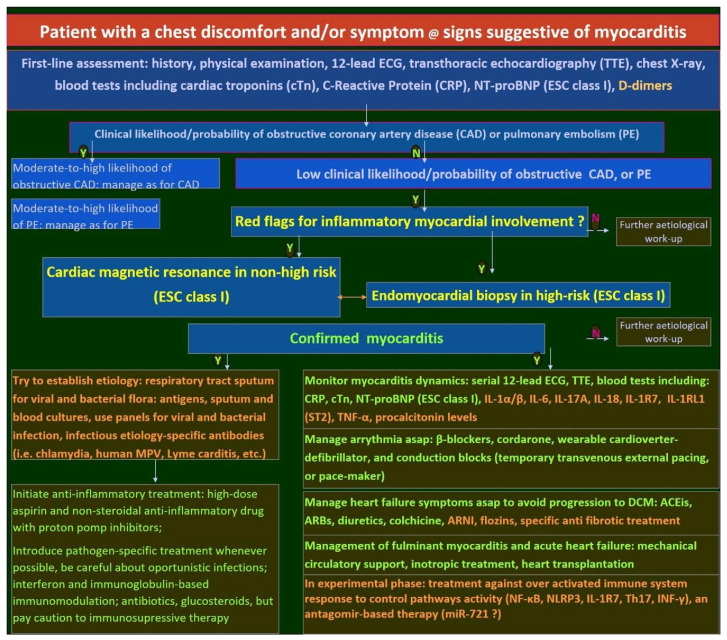
A proposed diagnostic and therapeutic algorithm for the management of patients presenting with chest discomfort and symptoms/signs suggestive of possible myocarditis, incorporating current ESC guidelines (in green color) [24]. In orange, the proposed development of the ESC guidelines algorithm. Red flags according to the ESC Guidelines include: recent or concomitant flu-like syndrome or gastroenteritis, infarct-like chest pain, palpitations, HF symptoms, ECG changes, Ventricular arrhythmias (isolated, complex), syncope, haemodynamic instability, elevated markers of myocardial lesion (hs-Tn, CK-MB elevation), elevated markers of HF (NT-proBNP), abnormal wall motion, increased wall thickness and/or impaired systolic function on imaging, CMR imaging with myocardial edema and/or LGE. Abbreviations: asap, as soon as possible; ACEis, angiotensin converting enzyme inhibitors; ARB, Angiotensin Receptor Blocker; ARNI, Angiotensin Receptor-Neprilysin Inhibitor; NT-proBNP, N-terminal B-type Natriuretic Peptide; DCM, dilated cardiomyopathy; HF, heart failure; N, no; Y, yes.

Figure 1 illustrates an algorithm for the diagnostic and therapeutic management of patients presenting with chest discomfort and signs/symptoms suggestive of myocarditis, incorporating current guideline recommendations.

### 1.3. Natural Course of Myocarditis

The natural course of myocarditis varies greatly between individuals, ranging from rapid and full recovery to the need for heart transplantation (HTx) in the most severe cases of dilated cardiomyopathy (DCM) and HF [2,4,7]. Aside from fulminant myocarditis, it is extremely difficult to predict at symptom onset who will deteriorate and who will recover quickly [32,33]. Myocarditis is a complex condition, with viral etiology playing a central role [1,2,3]. Standard treatment includes bed rest, management of the primary infection (antiviral or antibacterial therapy), and prevention and treatment of HF [23]. Treatment strategies may involve: no specific therapy, only symptomatic management (infusions, antipyretics), antibiotics, HF and antiarrhythmic therapy with beta-blockers, or a combination of optimal HF therapies such as ACE inhibitors, angiotensin II receptor blockers (ARBs), mineralocorticoid receptor antagonists (MRAs), diuretics, and other agents [5,7,23]. Beta-blockers should be considered for all patients with myocarditis due to their antiarrhythmic properties, which help prevent ventricular arrhythmias. Antibiotics are recommended only in cases of confirmed active bacterial infection, as viral infection is the usual cause of myocarditis.

Complicated myocarditis occurs in approximately 4% to 15% of cases, and around 1.2% of patients require durable mechanical circulatory support (MCS) [34,35]. The estimated mortality rate for acute myocarditis ranges from 1% to 7% [36,37,38]. In a Korean study involving nearly 3000 patients, 30-day all-cause mortality was 6.6%, and was independently associated with complicated disease course (HR: 13.92, 95% CI: 8.43–22.97), diabetes (HR: 3.99, 95% CI: 1.79–8.92), connective tissue disease (HR: 2.90, 95% CI: 1.25–6.74), and concomitant malignancy (HR: 7.64, 95% CI: 3.04–19.17) [35]. In complicated cases, myocarditis may progress to LV dysfunction, end-stage chronic HF, and DCM, requiring assist devices and/or HTx. In the Lombardy Registry, a multicenter registry of patients with acute myocarditis, cardiac mortality and HTx rates were 11.3% and 14.7%, respectively, in patients with complicated presentation, and 0% in uncomplicated cases (log-rank *p* < 0.0001) [36]. In contrast, multicenter data showed that among 419 patients admitted with fulminant myocarditis, 322 (77%) required temporary MCS upon intensive care unit admission, and one-third progressed to HTx or required ventricular support with LVAD/BiVAD during hospitalization [39]. Additionally, among 45,941 patients on the HTx waiting list in the Society for Heart and Lung Transplantation Registry, myocarditis was the primary diagnosis in 299 (0.7%) patients [34].

### 1.4. Elimination of Pathogen vs. Progression to Chronic Active Inflammation

The long-term outcome of myocarditis survivors varies greatly. Most acute myocarditis cases resolve within a few days. However, when active inflammation persists for more than three months from onset, it is defined as chronic active myocarditis [38]. The residual risk associated with acute myocarditis was identified in the Danish national cohort [40]. Although pathogen elimination and healing from myocarditis are possible in most cases, VMC can persist and remain active for months or even years [37]. In the Danish study, all-cause mortality was 16.9% over 8.5 years. Even younger patients who recovered from acute myocarditis without complications were at increased risk of HF and death compared to age- and sex-matched controls [39]. Similarly, data from a Korean cohort reported a mortality rate of 25.5% during a 10-year follow-up [35]. In the latter study, patients hospitalized for acute myocarditis received standard intensive care (43% required IABP, ECMO, mechanical ventilation, CRRT, or cardiopulmonary resuscitation) and typical cardiovascular medications, including ACE inhibitors, angiotensin II receptor blockers, norepinephrine, inotropes, intravenous nitrates, diuretics, antiarrhythmics, MRAs, beta-blockers, and/or digoxin [33].

However, the management, considered the “standard of care,” does not necessarily lead to complete recovery or full elimination of viral or bacterial material from cardiac cells [38]. The virus can persist in cardiac tissue, resulting in localized chronic myocarditis. Simultaneously, the innate immune system may remain activated, contributing to ongoing inflammation and myocardial fibrosis, which may eventually progress to DCM [38,41]. Both processes can occur simultaneously. Despite intensive medical treatment, some patients require LV assist devices to enable cardiac recovery; if this fails, HTx may be necessary for survival [36].

Administration of antiviral, antibacterial, and anti-inflammatory medications appears to reduce mortality rates and should be prioritized in both the acute and chronic phases of myocarditis management [1,3,7,37,38]. Addressing infection-induced inflammation remains a major challenge. First, diagnosing the underlying etiology can be difficult. Second, even when the virus or bacteria have been neutralized, autoimmune mechanisms may persist and impede recovery. Among the two main mechanisms preventing myocardial recovery, excessive activation of inflammasomes and cardiac fibrosis come to the fore [39]. Recently, new therapies directly targeting inflammatory pathways and capable of preventing cardiac fibrosis have been evaluated. Some of these have entered randomized clinical trials (RCTs).

The following case of a 64-year-old woman highlights the clinical challenges faced by healthcare providers in managing a patient hospitalized with myocarditis. In this article, we aim to discuss: (1) the human immune defense mechanisms against infection; (2) the role of pyroptosis and excessive immune response in developing complications and chronic active myocarditis; and (3) the potential role of emerging medications in myocarditis management.

## 2. An Illustrative Case Report

A 64-year-old woman with a significant medical history—including hypertension, dyslipidemia, hypothyroidism, nicotine dependence, degenerative joint disease, and chronic venous insufficiency—presented to the Emergency Department with several days of upper respiratory tract symptoms. She reported sore throat, chest pain during coughing, generalized musculoskeletal pain and stiffness, and subjective fever.

Initial laboratory work-up on admission to the cardiac unit revealed signs of viral infection with elevated inflammatory markers: procalcitonin (PCT) 1.27 ng/mL (reference < 0.5 ng/mL), C-reactive protein (CRP) 98.7 mg/L (reference < 5.0 mg/L), and interleukin-6 (IL-6) 19.2 pg/mL (reference < 7.0 pg/mL). BioFire nasopharyngeal swab tested positive for human metapneumovirus (hMPV). Cardiac biomarkers were markedly elevated: NT-proBNP 6978 pg/mL (reference < 125 pg/mL), troponin 786 ng/L (reference < 14 ng/L), creatine kinase (CK) 343 U/L (reference < 170 U/L), and CK-MB 20 U/L (reference < 24 U/L) (Table 1), without the dynamic profile typical of acute coronary syndrome (ACS).

Transthoracic echocardiography showed globally reduced LV-EF of 45%, with hypokinesis of the mid and apical segments of the inferior wall, interventricular septum, and posterior wall, without pericardial effusion. The patient was admitted to the cardiac intensive care unit for further evaluation and treatment of suspected myocarditis. After consultation with an interventional cardiologist, coronary intervention was deferred.

**Figure 2:**
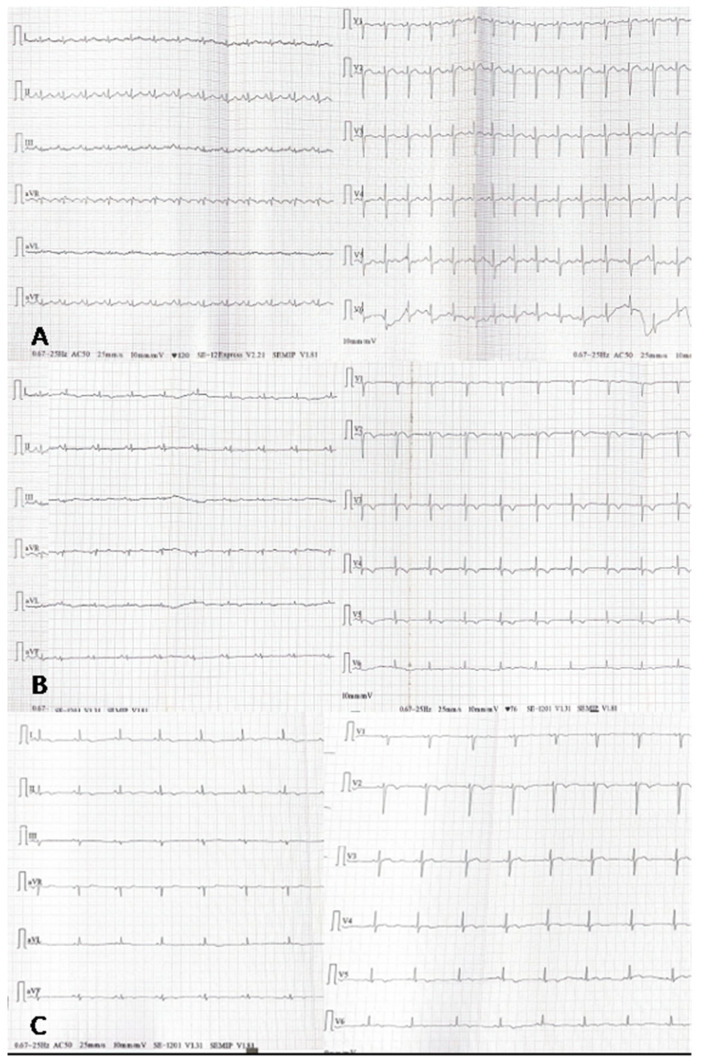
(**A**) A 12-lead ECG demonstrated sinus rhythm at 120 bpm, with normal axis and intervals and no significant ST-T abnormalities. (**B**) A 12-lead ECG showed new changes, including negative T waves in leads V2–V6. (**C**) At discharge, a 12-lead ECG showed sinus rhythm with a heart rate of 60 beats per minute, intermediate electrical axis, negative T waves in leads I and II, a flattened in aVF, and biphasic T waves in leads V4–V6.

On admission, the patient was conscious and hemodynamically stable. Physical examination revealed crackles in the lower fields of the right lung posteriorly and mid-axillary, and isolated basal crackles in the left lung. There was no peripheral edema. A 12-lead ECG demonstrated sinus rhythm at 120 bpm, with normal axis and intervals and no significant ST-T abnormalities (Figure 2A). Lung ultrasound revealed numerous confluent B-lines in the right lung’s lower posterior and mid-axillary fields, and isolated B- and A-lines in the scapular and paravertebral regions. The left lung showed isolated B-lines. No pleural effusion was observed.

**Figure 3:**
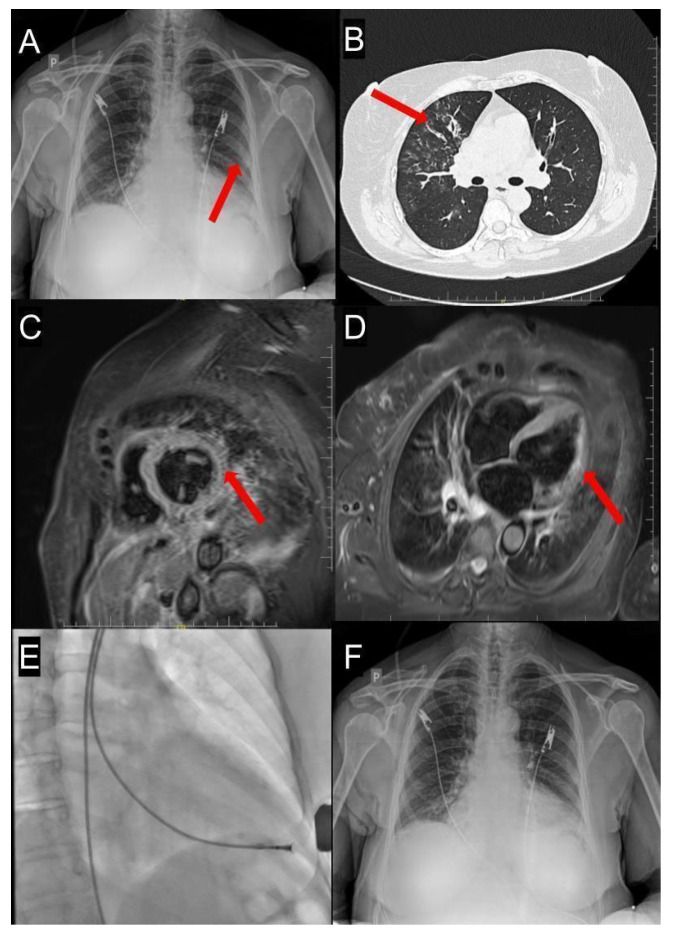
(**A**) Initial chest X-ray showing inflammatory changes consistent with pneumonia (indicated by the red arrow). (**B**) High-resolution chest CT (HRCT) showing bilateral inflammatory changes with a characteristic “tree-in-bud” pattern (marked by the red arrow). (**C**) Cardiac magnetic resonance (CMR) in a SAX view demonstrating generalized myocardial edema with late gadolinium enhancement (LGE) highlighted by the red arrow. (**D**) CMR in a four-chamber view demonstrating generalized myocardial edema with late gadolinium enhancement (LGE) highlighted by the red arrow. (**E**) Fluoroscopic image of a left ventricular muscle biopsy from the apical region, performed under echocardiographic guidance. (**F**) Follow-up chest X-ray at discharge showing a gradual resolution of the inflammatory changes.

Given her smoking history and occupational exposure to infections (working with children), a chest X-ray was performed (Figure 3A), which showed signs of concurrent pneumonia. Extensive microbiologic and immunologic testing (blood and urine cultures, and serologic IgA/IgG for *Chlamydia pneumoniae*, IgG for *Bordetella pertussis*, and *Mycoplasma pneumoniae*) yielded positive serum results. However, sputum PCR did not confirm bacterial presence, raising suspicion for atypical pneumonia.

Empirical antibiotic therapy was initiated with sulfamethoxazole/trimethoprim, followed by clarithromycin. On day 3, transthoracic echocardiography revealed progressive LV-EF decline to 20–25%, pericardial effusion of 1.2 mm, and generalized hypokinesis, particularly in the anterior wall and septum. A 12-lead ECG showed new changes, including negative T waves in leads V2–V6 (Figure 2B). Coronary CT angiography excluded ischemic causes of reduced LV-EF. HRCT confirmed bilateral “tree-in-bud” opacities consistent with atypical pneumonia (Figure 3B). Cardiac magnetic resonance (CMR) indicated probable myocarditis (Figure 3C,D).

HF therapy was initiated, including an SGLT2 inhibitor, beta-blocker (for rate control), ACE inhibitor, and eplerenone. On day four, the ACE inhibitor was replaced by sacubitril/valsartan (Sac/Val), an angiotensin receptor–neprilysin inhibitor (ARNI), at a daily dose of 24/26 mg.

Laboratory parameters improved from day 4. CRP decreased to 45 mg/L, CK to 247 U/L, troponin from 895 ng/L to 435 ng/L, and PCT to 0.43 ng/mL, although IL-6 increased to 59.7 pg/mL (Table 1).

Given the complete clinical and diagnostic picture and after obtaining informed consent, a left ventricular endomyocardial biopsy was performed (Figure 3E). During tissue sampling, the patient experienced angina progressing to cardiogenic shock with hypotension and tachycardia. Echocardiography revealed pericardial effusion without tamponade. Management included fluid resuscitation, dobutamine and norepinephrine infusions (5 mL/h and 2 mL/h, respectively), and protamine sulfate.

In the following days, the patient’s condition worsened with cardiorespiratory failure. Passive oxygen therapy and escalating vasopressors (up to 10 mL/h and 7 mL/h) were required. Laboratory results showed declining hemoglobin and glomerular filtration rate (GFR) down to 17 mL/min, necessitating therapy adjustments. From day 7 of hospital admission, a new increase in inflammatory markers was observed: CRP rose to 275 mg/L, IL-6 to 156 pg/mL, whereas PCT decreased to 0.11 ng/mL. Markers of myocardial necrosis continued to fall: CK 146 U/L, troponin 115 ng/L (Table 1). Repeated microbiological tests revealed a new *Streptococcus pneumoniae* infection, complicating the prior hMPV infection. Corticosteroids and azithromycin were initiated. The patient underwent cardiopulmonary rehabilitation. Vasopressors were gradually tapered, oxygen therapy was weaned, and heart failure therapy resumed. Due to hypotension, Sac/Val was withheld.

The echocardiographic examination performed on day 16 showed gradual improvement of LVEF to 60% and regression of pericardial effusion (from 1.5 cm to 0.5 cm). At discharge, a 12-lead ECG demonstrated regular sinus rhythm with a heart rate of 60 beats per minute, intermediate electrical axis, negative T waves in leads I and II, flattened T wave in aVF, and biphasic T waves in leads V2, V4–V6 (Figure 2C). Final laboratory tests showed normalization of necrosis markers (troponin 21 ng/L, CK-MB 9 U/L, CK 15 U/L), improved renal function (eGFR 97 mL/min), low inflammatory biomarkers (IL-6 9.0 pg/mL, CRP 10 mg/L, PCT < 0.05 ng/mL), and full resolution on chest X-ray (Figure 3F).

Clinically, the patient was NYHA class II; however, NT-proBNP remained elevated (3017 pg/mL) at discharge. The patient was prescribed bisoprolol 2.5 mg o.d., ramipril 5 mg o.d., dapagliflozin 10 mg o.d., eplerenone 25 mg o.d., and prednisone 20 mg o.d. with a tapering schedule.

**Figure 4:**
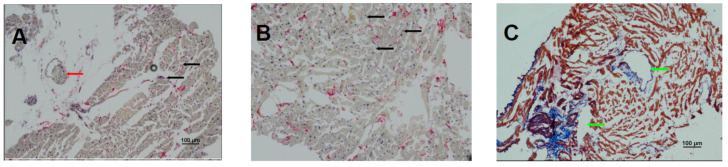
(**A**) DR.6 There is an increased expression of HLA class II (DR) (red arrow) antigens compared to the control, graded as 2+ on interstitial cells and coronary microvessels. On the left side, at the 9 o’clock position, a telescoped vessel (asymmetric thickening) is observed, while at the 8 o’clock position, a vasculopathic vessel is present. (**B**) CD68.1 Numerous CD68-positive macrophages (histiocytes) are present (black arrow). The macrophages are markedly activated and exhibit cytolytic activity toward injured cardiomyocytes to which they adhere. (**C**) MAS.3 Masson’s trichrome staining demonstrates the presence of connective tissue and necrotic changes in cardiomyocytes. Mild fibrosis is noted, predominantly perivascular. At the 7 o’clock position, there is a thickened (hypertensive-type) vessel (green arrow). The interstitium shows marked edema and pronounced cellular infiltration.

Histopathology confirmed focal necrosis, adipose infiltration, microvasculopathy with vascular smooth muscle cell (VSMC) proliferation, and CD68+ macrophage-mediated cardiomyocyte damage, consistent with myocarditis (Figure 4A–C). The biopsy indicated progression to chronic active myocarditis (CD68+) [42]. The patient remains under close cardiology follow-up with echocardiographic monitoring and guideline-directed therapy for heart failure and chronic myocarditis.

At the first telephone follow-up, the patient reported clinical improvement, no signs of heart failure decompensation, and only mild, improving fatigue. HF medications were titrated to maximum tolerated doses, and ARNI was reintroduced. Follow-up echocardiography was scheduled for one month, with a control CMR at six months post-treatment.

## 3. Discussion and the Literature Review

The presented case represents, to our knowledge, the first reported instance of hMPV-induced myocarditis complicated by subsequent *Streptococcus pneumoniae* infection in a patient without immunodeficiency. After a severe clinical course, the patient achieved “full recovery,” as evidenced by normalization of LV-EF to 60% and resolution of inflammatory markers. However, cardiac biopsy revealed progression to chronic active myocarditis with myocardial fibrosis and latent viral presence, indicating incomplete viral clearance or persistent activation of innate immunity. Consistently, NT-proBNP remained >3000 pg/mL, highlighting the risk of irreversible chronic HF and DCM, potentially necessitating MCS or HTx.

This underscores the need for novel strategies in immune-mediated myocarditis to reduce chronic inflammation and adverse cardiac remodeling. Pro-inflammatory cytokines critical for host defense may, when unchecked, drive chronic active inflammation and represent potential pharmacological targets [41].

### 3.1. Myocarditis Caused by Human Metapneumovirus (hMPV) and Opportunistic Bacterial Streptococcus pneumoniae Infection: A Brief Literature Review

hMPV is a paramyxovirus first identified in 2001 [43]. It is now recognized as a common cause of respiratory tract infections [44]. However, cardiac involvement in hMPV infections is extremely rare [45]. Only a few case reports describe hMPV-associated myocarditis, typically in immunocompromised individuals. Makhlouf et al. reported a case of a 14-year-old girl with Burkitt leukemia who developed severe hMPV-induced myocarditis, diagnosed using real-time PCR and CMR [46]. The patient was successfully treated with intravenous immunoglobulins. Wang et al. described hMPV-induced myocarditis complicated by Klebsiella pneumoniae co-infection in a 68-year-old man with liver cirrhosis [47]. The patient rapidly deteriorated, developed septic shock, and required intensive care, including ECMO, IABP, vasopressors, and broad-spectrum antibiotics. hMPV infections are known to be more severe in patients with immunodeficiency syndromes, autoimmune diseases (e.g., lupus, rheumatoid arthritis), HIV, cancer, or those receiving chemotherapy or immunosuppressive therapies [45]. The exact way in which hMPV invades cardiomyocytes is not well-known. However, the G and the F proteins of hMPV play a crucial role in the initial stages of respiratory tract infection [48]. The first one facilitates viral attachment and entry into host cells, whereas the F protein of hMPV mediates viral–host membrane fusion, transcription, and subsequent infection [48]. Integrins and heparan sulfate proteoglycans (HSPGs) have been implicated in the fusion and internalization processes [49,50,51]. HSPGs are the same entry receptors for various viruses, such as respiratory syncytial virus (RSV), SARS-CoV-2, human immunodeficiency virus (HIV), and herpes simplex virus (HSV) [48,50].

The G and F proteins are involved in modulating the host immune response, evading immune surveillance [52]. These functions enable hMPV to infect host cells more efficiently and replicate successfully. hMPV avoids host immune response and apoptosis due to the small hydrophobic (SH) protein of hMPV [53,54]. Studies suggest SH protein may be a viroporin forming ion channels [55]. It is believed to regulate host immune response through the NF-kB and the NLRP3 inflammasome [55]. hMPV activates NLRP3 inflammasome via its SH protein [55]. The contribution of NLRP3 inflammasome in the pathogenesis of RNA viruses occurs through its role as a trigger of inflammation, or both inflammation and viral replication [56,57].

Host immune factors can either promote or inhibit hMPV infection and replication. Among host restriction factors, leading to virus elimination, are cellular proteins that inhibit viral replication and spread [48,49]. The key host restriction factors for hMPV include (1) interferon-induced transmembrane proteins (IFITMs) that prevent viral entry and membrane fusion; (2) retinoic acid-inducible gene I (RIG-I); and (3) melanoma differentiation-associated gene 5 (MDA5) that activate NF-kB and IRF transcription factors [48,49,58,59,60]. Whereas, several host-promoting factors facilitate hMPV infection and replication within the host, including toll-like receptor 4, and -7 (TLR4), protease-activated receptor 1 (PAR-1), and transmembrane protease, serine 2 (TMPRSS2) [61,62,63]. TLR 4 and TLR 7 reduce infiltration of inflammatory cells, facilitate viral entry, and alter endosomal conditions. The PAR-1 promotes viral replication, while TMPRSS2 facilitates viral fusion.

Unfortunately, there is no solid treatment or vaccine for hMPV thus far; the lack of vaccines is a significant factor leading to a large number of hospitalizations during the epidemic season [64]. After many attenuated vaccines failed due to insufficient attenuation or poor protective effects, the subunit vaccine became the main focus of research [65]. Recently, Ma et al. presented a novel multi-epitope mRNA vaccine candidate to combat the HMPV virus [66].

In the present case, hMPV-induced myocarditis was complicated by a secondary *Streptococcus pneumoniae* infection a few days later. *Streptococcus pneumoniae* is known to translocate into the myocardium and form microlesions that impair cardiac function [18]. Pneumolysin (PLY), a pore-forming toxin produced by *S. pneumoniae*, is a key factor in this process [67]. Experimental studies have shown that microlesion formation requires the interaction of bacterial adhesin CbpA with the host laminin receptor and the bacterial cell wall with platelet-activating factor receptor (PAFR). Pneumococci utilize PAFR to cross barriers, such as from the lungs to the bloodstream, facilitating progression from pneumonia to bacteremia [68]. In phagocytic cells, cell wall components interact with toll-like receptor 2 (TLR) to activate NF-κB signaling and cytokine production. Surprisingly, no significant changes were detected in classic NF-κB target genes, including TNF-α, IL-1β, and I-κB, indicating that the signal was weak to activate the TLR-2-NF-κB pathway [68]. These findings suggest that *S. pneumoniae* invades cardiac tissue in a PAFR-dependent but TLR2-independent manner and impairs myocardial contractility without causing cardiomyocyte death [68]. Microlesion formation also required PLY [18]. Importantly, despite specific antibiotic treatment for Pneumococci, robust collagen deposition at former lesion sites, consistent with extensive scarring, is reported. This may explain adverse cardiac events during and after invasive pneumococcal disease [18]. Like in hMPV infection, a vaccination against *B. streptococcus* could protect the presented patient against cardiac complications [69].

### 3.2. Progression from Acute to Chronic Active Myocarditis

While acute immune activation is essential for host defense, excessive or prolonged responses may be harmful [70]. The possibility of myocarditis progressing from an acute to chronic phase, often with mixed etiology, is critical for patient management. It also highlights the need for targeted pathogen-specific treatment and understanding the variability of innate immune responses.

The immune system plays a central role in the pathogenesis and progression of cardiovascular disease (CVD) [70]. Macrophages are key players in the inflammatory cascade, secreting cytokines that fight pathogens through pyroptosis (a form of programmed cell death) while simultaneously recruiting other immune cells to the infection site [71,72]. Interleukin-1β (IL-1β) and IL-18 promote *T lymphocyte* activation and amplify cytokine and chemokine production [73]. Pathogen-specific immune responses converge on the activation of N-terminal gasdermin D (Nt-GSDMD), which forms pores in the cell membrane, disrupting membrane integrity, causing osmotic imbalance and leakage of intracellular contents—key features of pyroptosis [74]. In parallel, protein complexes cleave pro-caspase-1 to active caspase-1, which then processes pro-IL-1β and pro-IL-18 into their active forms IL-1β and IL-18 that are released during cell pyroptosis [75]. These cytokines amplify inflammation by recruiting more immune cells to the site [76]. IL-1 is linked to fever and leukocyte stimulation to release IL-6, IL-8, TNF-α, MCP-4, IP-10, CD40, RANTES (CCL5), and IL-18 [73]. These mediators further escalate the inflammatory response [77,78]. IL-18 is especially important in defending against intracellular pathogens via NK and Th1 cells, which produce interferon-γ (IFN-γ) [79]. Like IL-1, IL-18 is synthesized as an inactive precursor (pro-IL-18) that must be cleaved by caspase-1. Caspase-1 is an enzyme activated within the NLRP3 inflammasome complex, consisting of NLRP3, pro-caspase-1, and apoptosis-associated speck-like protein containing a caspase recruitment domain (ASC) [79,80].

**Figure 5:**
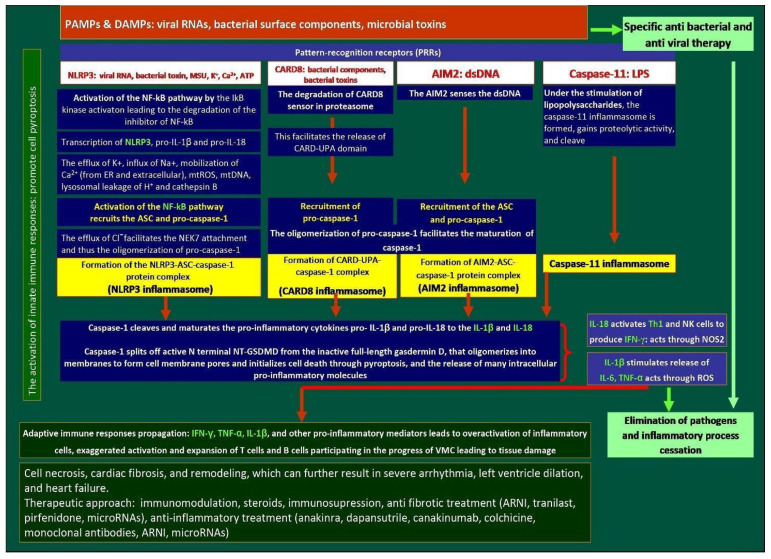
Pathways of innate response through inflammasome formation and potential targets for medical therapy (adopted from Xu et al. [72]). The key mechanisms by which inflammasomes oligomerize pro-caspase-1 into active caspase-1 and caspase-11, which facilitate GSDMD cleavage, along with IL-1β and IL-18 maturation. For detailed descriptions of inflammatory pathways, see the text. Abbreviations: AIM2, absent in melanoma 2; ARNI, angiotensin receptor-neprilysin inhibitor; ASC, apoptosis-associated speck-like protein containing a CARD; CARD8, caspase recruitment domain-containing protein 8; DAMPs, damage-associated molecular patterns; GSDMD, gasdermin D; IFN-γ, Interferon gamma; pro-IL-1b, pro-forms of interleukin 1b, IL-18; LPS, lipopolysaccharide; Nt-GSDMD, N-terminal gasdermin D; PAMPs, pathogen associated molecular patterns; dsDNA, double-stranded DNA; dsRNA, double-stranded RNA; NLRP, NOD-like receptor family pyrin domain; ROS, reactive oxygen species; TNF-α, Tumor Necrosis Factor alpha; VMC, viral myocarditis.

Caspase-1 can also be activated by other inflammasomes that belong either to the AIM2-like receptors, NOD-like receptors, or the TRIM family, which contain PYD or CARD domains [81,82]. In the presence of IL-12, IL-18 activates Th1 cells, macrophages, NK cells, NKT cells, B cells, DCs, and naive T cells to produce IFN-γ, essential for clearing intracellular infections [83,84]. In the absence of IL-12, IL-18 and IL-2 induce Th2 cytokines in NK and NKT cells and stimulate IFN-γ secretion in CD3-activated Th1 cells [84]. IFN-γ is vital for microbial clearance via nitric oxide synthase 2 (NOS2) activation [85]. The pathways leading to cell death and cytokine release vary depending on the pathogen [86]. At least four major inflammasome pathways are currently recognized [87]. The key mechanisms by which inflammasomes oligomerize pro-caspase-1 into active caspase-1 and facilitate GSDMD cleavage, along with IL-1β and IL-18 maturation, are illustrated in Figure 5.

### 3.3. Role of Inflammasomes in Myocarditis

The innate immune system detects both microbial invaders and sterile danger signals via pattern recognition receptors (PRRs). PRRs are expressed on the cell surface, intracellular vesicles, and in the cytosol of monocytes, macrophages, neutrophils, mast cells, dendritic cells, and natural killer (NK) cells [88]. Microbial components detected by PRRs are termed pathogen-associated molecular patterns (PAMPs), while endogenous danger molecules are called damage-associated molecular patterns (DAMPs), often arising from tissue damage or oxidative stress [89]. PRRs are classified into several major families based on protein domain homology: toll-like receptors (TLRs), NOD-like receptors (NLRs), C-type lectin receptors (CLRs), absent in melanoma 2 (AIM2)-like receptors, retinoic acid-inducible gene-I (RIG-I)-like receptors, cyclic GMP-AMP synthase (cGAS)–stimulator of interferon genes (STING), and Pyrin [90]. TLRs and CLRs are transmembrane receptors, while NLRs are cytoplasmic [89].

Among NLRs, NLR family pyrin domain containing 3 (NLRP3) is the best studied in relation to myocarditis [90]. The NLRP3 pathway has also been implicated in various inflammatory conditions, including atherosclerosis, Alzheimer’s disease, gut microbiota dysbiosis, and skin disorders such as urticaria [91,92,93,94,95,96]. Upon recognizing PAMPs or DAMPs, certain PRRs oligomerize and assemble with other proteins to form an inflammasome. A central component in this process is pro-caspase-1, which is activated via oligomerization to caspase-1 in mechanisms involving the NLRP3 pathway [97]. Other pathways include direct activation by cytosolic lipopolysaccharide (LPS) from Gram-negative bacteria, which activates caspase-11 (and its human homologs, caspase-4 and -5) [98].

#### 3.3.1. The NLRP3 Pathway

The canonical NLRP3 pathway requires two steps: priming and activation (Figure 5). Priming begins with pathogen recognition by pyrin domain-containing PRRs such as NLRP3. Recognition of stimuli like viral RNA, bacterial toxins, drugs, K+ efflux, Ca^2+^ mobilization, ER stress, lysosomal rupture, or mitochondrial dysfunction primes NLRP3. Activation involves NF-κB pathway stimulation, which is initiated by degradation of the NF-κB inhibitor via IκB kinase [99,100]. Activated NF-κB leads to recruitment of ASC and pro-caspase-1 [101]. Cl^−^ efflux enables NEK7 attachment, facilitating pro-caspase-1 oligomerization and forming the NLRP3-ASC-caspase-1 complex, known as the NLRP3 inflammasome. Caspase-1 has dual roles: (1) it cleaves and activates pro-inflammatory cytokines IL-1β and IL-18; (2) it processes full-length gasdermin D (GSDMD) into N-terminal (NT-GSDMD) and C-terminal fragments. NT-GSDMD forms membrane pores, initiating pyroptosis and cytokine release, including TNF-α and further NF-κB activation [94].

NLRP3 inflammasome activation has been demonstrated in myocarditis induced by CVA16, CVA10, and encephalomyocarditis virus (EMCV) [91]. EMCV-induced myocarditis relies on viral protein 2B, which promotes Ca^2+^ flux from the ER and Golgi into the cytoplasm, alongside K^+^ efflux [102,103,104]. SARS-CoV-2 enters cardiomyocytes via its spike protein binding to ACE2 with TMPRSS2, potentially activating NLRP3 inflammasomes and promoting inflammation [105,106].

#### 3.3.2. CARD8 Pathway

In the CARD8 pathway, bacterial components induce proteasomal degradation of the CARD8 sensor, releasing the CARD-UPA domain (Figure 5). This domain recruits and activates pro-caspase-1, forming the CARD8 inflammasome. Caspase-1 then processes pro-IL-1β, pro-IL-18, and GSDMD, similar to the NLRP3 pathway [107].

#### 3.3.3. AIM2 Pathway

AIM2 detects cytosolic double-stranded DNA (dsDNA), leading to ASC and pro-caspase-1 recruitment and inflammasome formation (Figure 5). Activated caspase-1 then processes IL-1β and IL-18, and cleaves GSDMD. The NT-GSDMD fragment forms membrane pores, facilitating cytokine release and pyroptosis [107].

#### 3.3.4. Caspase-11 Inflammasome

In mice, caspase-11 (and human caspase-4 and -5) directly bind cytosolic LPS from Gram-negative bacteria such as *Escherichia coli*, *Salmonella typhimurium*, *Shigella flexneri*, and *Burkholderia thailandensis* (Figure 5) [107]. When mice are infected by Gram-negative bacteria, LPS directly binds to the CARD of caspase-11, thus activating caspase-11. The activation process of human caspase-4 and caspase-5 is the same as that of caspase-11 [107]. Inflammatory caspases-4/11 can directly bind the lipid A moiety of LPS. However, how LPS, sequestered in the membranes of cytosol-invading bacteria, activates non-classical caspases remains not fully understood. In summary, in this mechanism under the stimulation of LPS, the caspase-11 inflammasome is formed, gains proteolytic activity, and cleaves [107].

### 3.4. Post-Infectious Phase

The exact mechanisms underlying hMPV-induced myocarditis remain unclear [108]. Current evidence suggests it results from both direct viral invasion and immune-mediated mechanisms [45,47]. Immune evasion by the virus enables persistence and tissue damage. Altered immune responses may contribute via molecular mimicry or bystander activation [109,110,111]. In addition, the patient in this case was a regular smoker (one pack/day), which may have contributed to endothelial injury and HF progression [112]. Nicotine upregulates NLRP3 expression [113], promotes monocyte adhesion, and foam cell formation. After endothelial dysfunction, monocytes adhere to vascular lesions, differentiate into macrophages, and engulf lipoproteins like oxLDL and ChCs [114]. These macrophages become foam cells, which promote chronic inflammation [115,116].

### 3.5. Therapeutic Approaches in VMC Based on Pathogen and Disease Phase

During early acute myocarditis, the innate immune response facilitates pathogen clearance. At this stage, targeting viral or bacterial agents is essential. However, if the inflammatory response persists, myocarditis can evolve into chronic active myocarditis—a detrimental condition requiring more comprehensive immune-modulating strategies [116].

VMC typically begins with the infection of cardiomyocytes by cardiotropic viruses such as enteroviruses (e.g., CVB3), adenoviruses, or parvovirus B19 [113]. Viral entry into cardiomyocytes is mediated by specific cellular receptors such as the coxsackievirus and adenovirus receptor (CAR). Once inside the cell, viral replication causes direct cytopathic effects and the release of viral proteins and nucleic acids, which are recognized as PAMPs. These activate PRRs, mainly toll-like receptors (TLRs), including TLR3, TLR4, and TLR7, present on cardiomyocytes and resident immune cells.

TLR activation triggers downstream signaling pathways, including NF-κB and interferon regulatory factors (IRFs), as well as the activation of the NLRP3 inflammasome. This promotes the maturation and secretion of IL-1β and IL-18, and upregulates other pro-inflammatory cytokines and chemokines such as TNF-α, IL-6, IFN-α, and IFN-β [117]. These mediators recruit innate immune cells (macrophages, neutrophils) into the myocardium. As inflammation progresses, dendritic cells present viral antigens to CD4+ and CD8+ T lymphocytes. CD8+ T cells kill infected cardiomyocytes, while CD4+ T cells coordinate cytokine production and support B-cell-mediated antibody responses [118]. While this response is initially protective, sustained inflammation can cause myocardial injury, fibrosis, and progression to chronic cardiomyopathy or dilated cardiomyopathy (DCM) [119]. Thus, balancing effective viral clearance with limiting inflammation is crucial [120].

#### 3.5.1. Specific Antiviral Therapy in the Acute Phase of Myocarditis

Viruses commonly linked to myocarditis include adenoviruses, enteroviruses (CVA/B, echoviruses), parvovirus B19, human herpesvirus 6 (HHV6), Epstein–Barr virus, cytomegalovirus, HIV, hepatitis C virus, influenza A/B, MERS-CoV, SARS-CoV, and SARS-CoV-2 [121]. Most VMC is managed symptomatically or supportively. Some viruses, such as adenoviruses and enteroviruses, are readily cleared from cardiomyocytes, whereas others (e.g., parvovirus B19, HHV6, Epstein–Barr virus, cytomegalovirus) tend to persist, contributing to HF, DCM, and arrhythmias, depending on host immune function [122,123,124].

Specific therapy includes interferon and immunoglobulin-based immunomodulation. In a pilot study by Kuhl et al., IFN-β cleared enterovirus/adenovirus in 100% of patients and improved LV function in 68% [122]. IFN-β also reduced viral load in chronic myocarditis due to enterovirus, adenovirus, or B19V [123]. Intravenous immunoglobulin therapy (IVIG), with IgG, or polyvalent IgG/IgA/IgM, may improve survival in children and adults with VMC.

#### 3.5.2. Immunosuppression in Active and Chronic Active Myocarditis

Glucocorticoids are first-line therapy in hemodynamically unstable or fulminant myocarditis [125]. A “Life support-based comprehensive treatment regimen” includes MCS devices, ventilation, dialysis, and high-dose glucocorticoids (200–400 mg methylprednisolone daily for several days) and IVIG, avoiding cytotoxic agents [126]. Without glucocorticoids/IVIG, benefits such as nitric oxide promotion, myocardial edema reduction, and cardiomyocyte survival are diminished [127]. Cytotoxic agents alone did not improve survival in fulminant myocarditis, as shown in the Myocarditis Treatment Trial [127]. The application of cytotoxic drugs could only gradually downregulate the cytokine levels. Immunosuppressive agents used in chronic active myocarditis include prednisolone alone or in combination with azathioprine or cyclosporine [128]. While some trials showed improved LV/RV function, survival benefits varied [129,130,131,132,133]. Long-term mortality was high, and routine immunosuppression was not supported by the Myocarditis Treatment Trial [133].

### 3.6. Targeting Inflammasomes: A Future for Myocarditis

hMPV activates NLRP3 inflammasome via its SH protein [53]. Likewise, many other viruses activate infection through this pathway. Therefore, the blockade of IL-1β production by using NLRP3 inflammasome inhibitors or the inhibitors of cascade factors, eventually leading to activation of NLRP3, might be a novel potential strategy for the therapy and prevention of hMPV infection (Table 2).

#### 3.6.1. Inhibition of NF-κB Pathway

Inhibiting NF-κB is a potential therapeutic strategy, particularly in cancer [134]. Drugs like aspirin, salicylates, dexamethasone, and natural products including curcumin, resveratrol, and epigallocatechin gallate, suppress NF-κB activity [134,135,136]. However, it is important to note that targeting NF-κB indirectly, for instance through proteasome inhibition, carries a risk of off-target effects. Bortezomib (BTZ), Ixazomib, and Carfilzomib are proteasome inhibitors used in the treatment of multiple myeloma (MM) and other hematological malignancies; these agents block IκB degradation and thereby inhibit NF-κB activation. Despite their clinical efficacy, they have been associated with adverse cardiac effects, including arrhythmias, HF, and inflammatory complications such as pericarditis and myocarditis [137]. These observations highlight the delicate balance between therapeutic benefit and cardiotoxicity when modulating key inflammatory pathways such as NF-κB in oncology.

#### 3.6.2. Direct NLRP3 Inhibitors

Acute inflammasome activation is beneficial for infection defense but harmful when prolonged. Direct NLRP3 inhibition is thus a therapeutic option in chronic active myocarditis. In murine models of sepsis and LPS-induced cardiac injury, corticosteroids suppressed NLRP3 formation, cysteine asparaginase-1 activation, and IL-1β secretion [138]. This demonstrates that cortisone is a novel immunomodulatory factor with the ability to inactivate NLRP3 inflammasomes and protect the myocardium from septic injury. NLRP3 inhibition (e.g., MCC950) reduced arrhythmias and remodeling in myocarditis. In rats with myosin peptide-induced myocarditis (experimental group) treated with an NLRP3 inhibitor (MCC950; 10 mg/kg, daily for 14 days) for three weeks or left untreated (Table 2), MCC95 mitigated the myocarditis-induced leakage of Ca^2+^. This finding suggests that activation of CaMKII is crucial for the effects of myocarditis on RVOT cardiomyocytes [138].

#### 3.6.3. Colchicine

Colchicine has broad anti-inflammatory effects, and it is a potent inhibitor of tubule polymerization. Colchicine disrupts microtubules that are responsible for the subcellular transport of ASC and NLRP3 within macrophages and are necessary for proper cytosolic localization and activation of the NLRP3 inflammasome components. Recent studies suggest benefits in acute and chronic myocarditis (Table 2) [139,140,141,142,143]. In one study, colchicine reduced 90-day mortality, arrhythmia, and HF (193 [17.0%] vs. 279 [24.5%], log-rank *p* < 0.001; HR 0.66, 95%CI [0.55–0.79], respectively) [139]. Side effects were mild. Another study showed fewer recurrences (respectively, 19.2% vs. 43.8%; *p* = 0.001) and a longer event-free survival (*p* = 0.005) in 175 patients with idiopathic/viral etiology of myocarditis receiving colchicine [140]. Colchicine was well-tolerated, and colchicine-associated side effects were mild and occurred in 3 (1.7%) patients [140]. Pappritz et al. demonstrated that colchicine treatment significantly reduced cardiac inflammation and improved LV function in a murine model of autoimmune myocarditis [142]. As yet, the only published, randomized controlled trial (RCT) with colchicine in myocardial injury due to COVID-19 infection, the GRECCO-19 trial, showed that patients who received colchicine had significantly improved time to clinical deterioration [143]. In comparison, there were no significant differences in hs-Tn or CRP levels [143].

These findings support the hypothesis that targeting microtubule dynamics and inflammasome activation may translate into clinical benefit. Ongoing clinical trials, including NCT05855746 (Colchicine vs. placebo in acute myocarditis; https://clinicaltrials.gov/study/NCT05855746 accessed on 11 August 2025) and CMP-MYTHiC NCT06158698 (Colchicine in cardiomyopathy and myocarditis; https://cdek.pharmacy.purdue.edu/trial/NCT06158698/ accessed on 11 August 2025), aim to assess the efficacy and safety of colchicine in human myocarditis. Preliminary data and experimental results suggest that colchicine could become a valuable adjunctive therapy by reducing myocardial inflammation, limiting adverse remodeling, and potentially improving long-term cardiac outcomes.

#### 3.6.4. Dapansutrile (OLT1177)

Dapansutrile is an oral NLRP3 inhibitor tested in a phase 1B trial in 30 patients with stable HFrEF [144]. Improvements in LVEF [from 31.5% (27.5–39) to 36.5% (27.5–45), *p* = 0.039] and in exercise time [from 570 (399.5–627) to 616 (446.5–688) seconds, *p* = 0.039] were seen in the dapansutrile 2000 mg cohort (Table 2) [144]. It was well-tolerated and reduced IL-1β and IL-18, supporting its use in inflammasome-driven cardiac inflammation [144]. Dapansutrile’s anti-inflammatory effect is obtained by directly blocking the assembly and activation of the NLRP3 inflammasome complex. Thus, dapansutrile prevents downstream release of pro-inflammatory cytokines such as IL-1β and IL-18, which are implicated in cardiac inflammation and remodeling.

If other studies support dapansutrile efficacy, it is potentially a new drug for use in cardiovascular conditions associated with excessive inflammasome activation.

#### 3.6.5. INF200

INF200 is a selective oral NLRP3 inflammasome inhibitor, a novel small-molecule inhibitor structurally based on the 1,3,4-oxadiazol-2-one scaffold. In the experimental study by Wang et al. [145], it reduced inflammation, fibrosis, and cardiomyocyte apoptosis, and improved LV function (Table 2) [145]. These findings suggest that INF200 not only attenuates the inflammatory response but also protects against structural remodeling of the myocardium.

#### 3.6.6. Canakinumab

Canakinumab is a monoclonal antibody targeting IL-1β. It is approved by the U.S. Food and Drug Administration for the treatment of systemic juvenile idiopathic arthritis, active Still’s disease, and certain types of autoinflammatory periodic fever syndromes. In the CANTOS trial (*n* = 10,061), patients who received the intermediate canakinumab dose (150 mg) had a reduced primary endpoint and a composite of cardiovascular death, nonfatal acute MI, nonfatal stroke, and systemic inflammation (IL-6, CRP) (Table 2) [146].

#### 3.6.7. Anakinra and IL-1 Receptor Accessory Protein Monoclonal Antibody

Anakinra inhibits IL-1 receptor 1, thereby inhibiting both IL-1β and IL-1α. It is already approved for other inflammatory conditions [147]. In the MRC-ILA (Medical Royal Council InterLeukin-1 Antagonist) heart study, patients with non-ST-segment elevation ACS were randomized to daily administration of anakinra or a matching placebo for 2 weeks (Table 2) [148]. Anakinra reduced CRP and IL-6, but increased major adverse cardiac events at 1 year [148]. In the VCU-ART3 trial (Virginia Commonwealth University Anakinra Remodeling Trial 3), anakinra reduced HF incidence in STEMI patients when given within 12 h after the onset of symptoms [149].

Formerly published case reports of fulminant myocarditis showed the spectacular efficacy of IL-1 blockade with anakinra [150,151]. Promising results led to the ARAMIS trial investigating anakinra in acute myocarditis [152]. The pending ARAMIS trial is a multicenter, randomized, placebo-controlled study designed to assess the efficacy and safety of anakinra in patients with acute myocarditis. Patients receive anakinra for 14 days, with the aim of reducing myocardial inflammation by blocking IL-1α and IL-1β signaling. Preliminary results have shown a significant reduction in systemic inflammatory markers such as CRP and IL-6, along with encouraging trends toward improved LV function. While long-term clinical outcomes are still under investigation, the study supports the potential role of IL-1 blockade as a targeted therapy in acute myocarditis.

In addition, a recent study demonstrated an advantage of IL-1 receptor accessory protein (IL1RAP) blockade with a monoclonal antibody [153]. IL1RAP (also called IL1R3) is a shared subunit for the IL-1, IL-33, and IL-36 isoform receptors. An IL1RAP blockade might be a potential therapeutic strategy in viral and autoimmune myocarditis. IL1RAP blockade reduced inflammatory monocytes, T cells, neutrophils, and eosinophils in the heart in CVB3-mediated VMC in mice compared with placebo and IL1Ra (anakinra) treatment alone [153]. In conclusion, IL1RAP blockade reduced cardiac inflammation and disease severity in VMC and protected against cardiac dysfunction in autoimmune myocarditis, with higher efficacy compared with anti-IL-1 treatment alone.

#### 3.6.8. Monoclonal Antibodies and Drugs Targeting IL-18

Recently, a novel humanized monoclonal anti-human IL-1R7 antibody that specifically blocks and suppresses the inflammatory signaling of IL-18 was developed [154,155,156]. It acts by reducing IL-18-induced NF-κB and IFN-γ activation and IL-6 production in human cell lines. As yet, many researchers use IL-18 as a marker of inflammasome activation [156]. In recent studies, the strategy of blocking IL-1R7 in hyperinflammation in vivo using animal models was explored (Table 2) [154,155]. It is important to note that IL-1R7 is a potential virgin therapeutic strategy for the investigation of its clinical potential in treating IL-18-mediated diseases, as this area remains to be explored [154,155,156].

**Table 2:** Pharmacological strategies against excessive activity of inflammasomes. Inhibitors of NLRP3 and Interleukins: a novel therapeutic approach.

Study	Medication/Comparator	Type of the Study	Study Design	Main Findings	Outcomes	Remarks/Limitations
Inhibitors of the NLRP3 pathway						
Chin, CG et al. 2024 [138]	MCC950 vs. placebo	Experimental animal model	Rats with myosin peptide–induced myocarditis (experimental group) were treated with an NLRP3 inhibitor (MCC950; 10 mg/kg, daily for 14 days) or left untreated	Rats treated with MCC950 improved their LV-EF and reduced the frequency of premature ventricular contraction	Changes in heart structure may be mitigated by inhibiting NLRP3 signaling.	A study on animal models
Golino M et al. 2024 [139]	Colchicine vs. placebo in a 1:1 proportion	Retrospective multicenter study in the US of patients hospitalized with acute myocarditis	In total, 1137 patients with acute myocarditis treated with colchicine within 14 days of diagnosis vs. those not receiving colchicine	The incidence of the all-cause death was 3.3% vs. 6.6% (HR, 0.48, 95% CI, 0.33–0.71; *p* < 0.001), ventricular arrhythmias: 6.1% vs. 9.1% (HR, 0.65, 95% CI, 0.48–0.88; *p* < 0.01), and acute HF: 10.9% vs. 14.7% (HR 0.72, 95% CI, 0.57–0.91; *p* < 0.01) in patients treated with colchicine or not, respectively	Patients with acute myocarditis treated with colchicine within 14 days of diagnosis have better outcomes at 90 days	Short-term outcomes
Collini et al. 2024 [140]	45.1% of patients were treated with colchicine	Retrospective cohort study	A total of 175 patients with pericarditis and myocarditis, 88.6% with an idiopathic/viral etiology of myocarditis	In multivariable Cox regression analysis, women (HR 1.97, 95% CI 1.04 to 3.73; *p* = 0.037) and corticosteroid use (HR 2.27, 95% CI 1.15 to 4.47; *p* = 0.018) were risk factors of recurrence, and colchicine use prevented recurrences (HR 0.39, 95% CI 0.21 to 0.76; *p* = 0.005)	After a median follow-up of 25.3 (IQR 8.3–45.6) months, colchicine use was associated with a lower incidence of recurrences (respectively, 19.2% vs. 43.8%; *p* = 0.001) and a longer event-free survival (*p* = 0.005)	Concomitant pericarditis and myocarditis
Pappritz et al. 2022 [142]	Colchicine (5 µmol/kg p.o. daily) vs. vehicle (PBS)	Preclinical, experimental	Murine model of CVB3-induced myocarditis; treatment started 24 h post-infection for 7 days	Colchicine significantly improved LV-EF, reduced viral load, and decreased inflammatory cell infiltration (ASC^+^, caspase-1^+^, IL-1β^+^ cells) in myocardium and spleen.	Reduced fibrosis markers, cardiac troponin I, and lower collagen deposition.	Preclinical only
GRECCO-19 trial, 2020 [143]	52.4% of the total 105 patients were treated with colchicine	RCT phase 3	to explore the potential of colchicine to attenuate COVID-19–related myocardial injury	Patients who received colchicine had a significantly improved time to clinical deterioration. There were no significant differences in hs-Tn or CRP levels	a significant clinical benefit from colchicine in patients hospitalized with COVID-19	Cardiac complications of COVID-19 infection
NCT05855746 [https://clinicaltrials.gov/study/NCT05855746 accessed on 11 August 2025]	Colchicine vs. placebo	RCT phase 3	Three hundred adults with acute myocarditis; primary endpoint at 6 months: composite of rehospitalization, recurrent chest pain, arrhythmias, changes in LGE percentage by CMR	No results, as yet	Results expected post 2028	Results not yet available
CMP-MYTHiC NCT06158698 [https://cdek.pharmacy.purdue.edu/trial/NCT06158698/ accessed on 11 August 2025]	Colchicine vs. placebo	RCT phase 3	In total, 80 adults with chronic inflammatory cardiomyopathy and impaired LV-EF or ventricular arrhythmias were to receive colchicine or placebo for 6 months, with outcomes assessed by imaging, biomarkers, and arrhythmic burden.	No results, as yet	Results expected post 2026	Chronic myocarditis, small population
Wohlford GF et al. 2020 [144]	Dapansutrile (OLT1177)	RCT phase 1B	Patients with HFrEF, dose escalation, single-center, repeat dose safety and pharmacodynamics study of dapansutrile in stable patients with HFrEF	Improvements in LV-EF [from 31.5% (27.5–39) to 36.5% (27.5–45), *p* = 0.039] and in exercise time [from 570 (399.5–627) to 616 (446.5–688) seconds, *p* = 0.039] were seen in the dapansutrile 2000 mg cohort	Treatment with dapansutrile was well-tolerated and safe over a 14-day treatment period.	A study for HFrEF, not specifically in myocarditis
Wang et al. 2022 [145]	INF200 (1,3,4-oxadiazol-2-one)	preclinical	Experiments on heart stress in rats	INF200 works by inhibiting the NLRP3 inflammasome, which in turn reduces inflammation and its associated detrimental effects on the heart	Reduced cardiac biomarkers and ischemia–reperfusion injury in diet-induced metabolic heart stress in rats	A study on animal models
IL-1 receptor antagonists						
CANTOS TRIAL [146]	Canakinumab: IL-1 receptor antagonist given s.c. in doses of 50 mg, 150 mg, or 300 mg, vs. placebo	RCT phase 3	A total of 10,061 patients with prior MI and high hs-CRP (≥2 mg/L) given s.c. canakinumab at 50 mg, 150 mg, or 300 mg every 3 months	The 150 mg dose of canakinumab significantly reduced the incidence of recurrent cardiovascular events compared to placebo	The canakinumab dose of 150 mg was associated with a reduced occurrence of the primary endpoint and a reduction in IL-6 and CRP levels	A study for myocardial infarction, not specifically in myocarditis
MRC-ILA [148]	Anakinra given s.c. IL-1 ra vs. placebo, 1:1 allocation	RCT phase 2	182 patients with NSTEMI; treatment for 14 days; primary endpoint: 7-day CRP	Significant CRP reduction (≈50% vs. placebo); no difference in 30-day or 3-month MACE	Lower inflammation; no clinical benefit; unexpected rise in CV events at one-year follow-up	A study for myocardial infarction, not specifically in myocarditis
VCU-ART3 [149]	Anakinra 100 mg once daily or twice daily vs. placebo	RCT phase 2	99 STEMI patients within 12 h of symptom onset; 14-day treatment; primary endpoint: CRP level to day 14; follow-up to 12 months for echocardiographic remodeling and MACE	Not yet fully published, but early phase results showed significant CRP reduction during treatment	Primary: reduced inflammation (CRP AUC); secondary: pending data on LV remodeling and MACE over 12 months.	A study for myocardial infarction, not specifically in myocarditis
ARA-MIS Trial (Kerneis et al.) [152]	Anakinra (IL-1 receptor antagonist: IL-1 ra) 100 mg vs. placebo	RCT phase 2	120 patients hospitalized with CMR-proven acute myocarditis, without severe hemodynamic instability or cardiogenic shock	No significant difference from myocarditis complications within 28 days. Significant reduction in systemic inflammatory markers such as CRP and IL-6	Safety confirmed; well-tolerated, but no efficacy signal in low-risk patients	Short course, low-risk population, low incidence of complicated myocarditis in both groups
Lema et al. 2024 [153]	IL1RAP monoclonal antibody vs. placebo, or vs. anakinra/IL-1Ra	Preclinical, mice model	Induced CVB3-mediated myocarditis or experimental autoimmune myocarditis in mice, followed by the treatment with anti-mouse IL1RAP monoclonal antibody vs. placebo, or IL-1Ra treatment	IL1RAP blockade with a monoclonal antibody, compared with placebo and IL1Ra, reduced inflammatory monocytes, T cells, neutrophils, and eosinophils in the heart in CVB3-mediated VMC, and preserved heart function on echocardiography in autoimmune myocarditis	The effect on the reduction in inflammation was higher in IL1RAP blockade compared with anti-IL-1Ra treatment alone, and placebo	Study of viral and autoimmune myocarditis
Interleukin-18 inhibitors						
Li et al. 2021 [154]	Anti–IL-1R7 monoclonal antibody vs. isotype control	Preclinical, in vitro human cells + in vivo mouse models	Assessed suppression of IL-18–mediated signaling in human PBMCs and whole blood; in mice, evaluated protection against LPS- or Candida-induced hyperinflammation	Blockade of IL-1R7 strongly inhibited IL-18–induced NF-κB activation and downstream cytokines (IFNγ, IL-6, TNFα) in mice, significantly reduced systemic inflammation, and protected tissues (lung, liver) from LPS-induced injury	Demonstrated proof-of-concept that IL-1R7 blockade effectively attenuates IL-18–driven inflammation.	No direct myocarditis model was used
Jiang L et al. 2024 [155]	anti-human IL-1R7 antibody	Mice model	A novel humanized monoclonal antibody, which specifically blocks the activity of human IL-18 and its inflammatory signaling in human cells and whole blood cultures, was tested in hyperinflammation in the acute lung injury model	In the current study, anti-IL-1R7 suppressed LPS-induced inflammatory cell infiltration in lungs and inhibited subsequent IFN-γ production	An IL-1R7 antibody protects mice from LPS-induced tissue and systemic inflammation	Aimed to combat macrophage activation syndrome and COVID-19 infection

Abbreviations: ASC, apoptosis-associated speck-like protein containing a CARD; CMR, cardiac magnetic resonance; CRP, C-Reactive Protein; CVB3, Coxsackievirus B3; hs-CRP, high-sensitivity C-Reactive Protein; HFrEF, heart failure with reduced ejection fraction; hs-Tn, high-sensitivity troponin T; IFN-γ, interferon gamma; IL, interleukin; IL-1R7, interleukin-1 receptor 7; LGE, late gadolinium enhancement; LPS, lipopolysaccharide; LV-EF, left ventricular ejection fraction; MACE, major cardiac adverse events; NSTEMI, Non ST Elevation Myocardial Infarction; NLRP3, NLR family pyrin domain containing 3; PBMCs, peripheral blood mononuclear cells; s.c., subcutaneous; STEMI, ST Elevation Myocardial Infarction; VMC, viral myocarditis.

### 3.7. Angiotensin Receptor-Neprilysin Inhibitor (ARNI)

Sacubitril/valsartan (Sac/Val) is an angiotensin receptor-neprilysin inhibitor (ARNI) that has an established role in chronic HFrEF as it decreases the risk of death and hospitalization [157,158,159,160,161]. In RCT, Sac/Val reduced cardiovascular mortality (by 20%) and all-cause mortality (by 16%) compared to enalapril [161]. However, RCT excluded patients with ACS < 4 weeks, primary hypertrophic or infiltrative DCM, acute myocarditis, constrictive pericarditis or tamponade, active infection, planned LVAD, and other specific conditions [161,162,163]. Furthermore, data for Sac/Val use in patients in NYHA class IV and requiring inotropic therapy are limited [161]. In PARADIGM-HF and OUTSTEP-HF trials, only 0.7% of patients had New York Heart Association (NYHA) functional class IV symptoms [161,162]. The multicenter PIONEER-HF trial assessed the impact of Sac/Val on NT-proBNP and high-sensitivity cardiac troponin T (hs-TnT) levels, and HF hospital admissions in patients hospitalized for acute HF after achieving HF stabilization [163]. A greater reduction in NT-proBNP and hsTnT, and a lower rate of rehospitalization for HF, were observed in the Sac/Val arm compared to the enalapril arm at 4 and 8 weeks (Table 3). The LIFE trial, which was scheduled to assess Sac/Val in patients with HFrEF and recently advanced HF (NYHA class IV), has not randomized a predefined group of 400 patients, as it was terminated prematurely due to the COVID-19 pandemic [164]. The results of the LIFE trial in a group of 335 patients showed that Sac/Val was not superior to valsartan and had a 29% discontinuation rate during the 24 weeks of the trial [164,165]. Compared to valsartan, treatment with Sac/Val has not improved the clinical composite of number of days alive, out of hospital, and free from HF events [165].

#### 3.7.1. Off-Label Use of ARNI

There is no evidence of benefits from the prescription of Sac/Val in patients with acute HF, non-ischemic cardiomyopathy, such as chemotherapy-induced DCM, or acute myocarditis [166]. Despite limited data, clinicians often prescribe Sac/Val based on its beneficial mechanisms, such as enhancing bioavailability of circulatory and myocardial nitric oxide, which leads to an increase in cyclic guanosine monophosphate (cGMP) and the activation of the protein kinase G, reducing fibrosis and inflammation [167,168,169]. CMR studies show reduced LV scarring, improved LV contractility, and faster recovery [170].

#### 3.7.2. ARNI in Acute Myocardial Infarction: RCT Results

In patients who suffered from MI, observational studies indicated that use of ARNI might be superior compared with the use of ACE inhibitors or ARBs alone (Table 3) [171]. However, in RCT, the use of ARNI in patients with acute MI was not associated with a significantly lower incidence of death from cardiovascular causes or incident HF compared to ramipril [172]. The PARADISE-MI trial was a large randomized study evaluating the effects of Sac/Val compared to ramipril in patients following acute MI with reduced LV-EF (≤40%) and signs of HF or pulmonary congestion. A total of 5661 patients underwent randomization within 7 days post-MI; 2830 were assigned to receive Sac/Val and 2831 to receive Ramipril [172]. Over a median of 22 months, a primary outcome (cardiovascular death, HF hospitalization, or outpatient HF) occurred in 338 patients (11.9%) in the Sac/Val group and in 373 patients (13.2%) in the ramipril group (NS). However, compared to ramipril, Sac/Val showed a trend toward fewer HF events and a lower incidence of hypotension.

#### 3.7.3. ARNI in Doxorubicin-Induced DCM

Doxorubicin, an anthracycline chemotherapeutic agent used in various cancers, is well-known for its dose-dependent cardiotoxicity leading to DCM. Established doxorubicin cardiomyopathy is often fatal, with mortality reaching approximately 50% after the onset of HF [173]. Therefore, patients receiving doxorubicin should be closely monitored with regular HF symptom assessment, echocardiography, and biomarkers such as NT-proBNP, as DCM is typically irreversible once it occurs. Currently, there is no effective treatment for established doxorubicin-induced cardiomyopathy. This is due to the complex mechanisms of doxorubicin cardiotoxicity, including ROS generation, NLRP3 inflammasome activation, IL-1β secretion, pyroptosis, matrix metalloproteinase activity, mitochondrial dysfunction, apoptosis, and autophagy of cardiomyocytes, resulting in progressive LV dilation and systolic dysfunction [174,175,176]. It is postulated that both doxorubicin-related cardiac toxicity can be attenuated by Sac/Val administration [177,178]. Experimental studies have shown promising results with Sac/Val. In rodents, ARNI therapy reduced cardiac toxicity and fibrosis more effectively than valsartan alone, and this benefit was associated with modulation of matrix metalloproteinase activity [177]. In Sac/Val-treated rats, an attenuated histological evidence of cellular toxicity and fibrosis was found [177]. In mice, Sac/Val attenuated inflammation, fibrosis, and apoptosis while promoting autophagy via the AMPKα–mTORC1 pathway [179]. Dindas et al. also showed that Sac/Val reduced oxidative stress and caspase-3 activation (Table 3) [180]. Moreover, combined low-dose ARNI with SGLT2 inhibitors showed the highest survival rates and minimal toxicity in mouse models [181]. The survival rate of acute doxorubicin-injected mice in treatment groups including SGLT2i only, ARNI only, and ARNI/SGLT2i combination increased to 66.7%, 50%, and 66.7%, respectively, from 25% in the doxorubicin + vehicle group [182]. Of note, the low-dose ARNI/SGLT2i group showed the highest survival (85.7%), whereas the high-dose ARNI was associated with high cardiac toxicity [182]. Also, a recent study showed that a low dose of Val/Sac leads to successful reversal of acute HF in chemotherapy-induced cardiomyopathy [183].

These findings suggest translational potential for ARNI therapy in myocarditis, which shares similar inflammatory and fibrotic pathways with doxorubicin cardiotoxicity.

#### 3.7.4. Potential of ARNIs in Acute Myocarditis: A Review of the Literature

Although ACE inhibitors remain foundational in HF treatment, ARNIs such as Sac/Val are increasingly used when ACE inhibitor therapy is insufficient [184,185,186]. In acute myocarditis, Sac/Val has shown superior outcomes in improving LV contractility and reducing dilation compared to ACE inhibitors. Nesukay et al. demonstrated improved outcomes with Sac/Val compared to ACE inhibitors after six months treatment in patients with reduced initial LV-EF ≤ 40% (β = 0.601; *p* = 0.016), decreased longitudinal and circular global LV systolic deformation of ≤8.5% (β = 0.687; *p* = 0.012) and ≤9.0% (β = 0.611; *p* = 0.024), respectively, LV dilatation with end-diastolic volume index ≥ 102 mL/m^2^ (β = 0.712; *p* = 0.006), NYHA III or higher functional class (β = 0.425; *p* = 0.047), the presence of LV delayed enhancement in at least five segments or more according to CMR data (β = 0.548; *p* = 0.031) (Table 3) [187].

The anti-inflammatory effects of Sac/Val have been further elucidated in the study by Liang et al., who demonstrated that, beyond its established role as a neoprilysin inhibitor and angiotensin receptor blocker, Sac/Val inhibits the NLRP3, thus IL-1β–mediated inflammatory pathway in the myocardium [188]. In experimental models, treatment with Sac/Val reduced levels of key inflammatory cytokines, including IL-6 and TNF-α, and attenuated cardiac fibrosis and adverse remodeling [189]. Of note, Zile et al. reported that aldosterone, ST2 (soluble tumorigenicity suppressor 2, a receptor from the interleukin-1 family associated with cardiac remodeling and fibrosis), TIMP-1 (metallopeptidase inhibitor 1), MMP-9, PINP (aminoterminal propeptide of type I collagen), and N-terminal propeptide of procollagen type III had decreased more in the Sac/Val group than in the enalapril group eight months after randomization [190]. Liang et al. demonstrated that Sac/Val alleviates myocarditis by inhibiting Th17 cell differentiation independently of the NLRP3 inflammasome pathway [188]. Reduction in levels of all aforementioned biomarkers might have a beneficial pleiotropic effect in myocarditis, reducing fibrosis, inflammation, and arhythmogenesis.

These anti-inflammatory properties support Sac/Val’s role in mitigating myocardial inflammation and improving long-term cardiac function following injury.

### 3.8. Role of Cardiac Fibrosis and Anti-Fibrotic Treatment Approaches

Cardiac fibrosis is a maladaptive process marked by excessive extracellular matrix (ECM) deposition, primarily of collagens I and III [191,192]. This increases myocardial stiffness, reduces compliance, and impairs systolic and diastolic function, contributing to HF progression. In clinical practice, the effect of cardiac fibrosis can be observed on CMR and biochemical markers of HF [193,194]. Fibrosis arises from interactions among inflammatory, neurohormonal, molecular, and profibrotic signaling pathways [195,196,197]. Injury or chronic stress activates immune cells (macrophages, mast cells), which secrete pro-inflammatory cytokines (IL-1β, IL-6, TNF-α), promoting fibroblast activation. A pivotal mediator of fibrogenesis is transforming growth factor-beta (TGF-β), which promotes the differentiation of resident cardiac fibroblasts into activated myofibroblast cells that secrete large quantities of ECM components. TGF-β signals through canonical Smad-dependent pathways and alternative non-canonical pathways, leading to transcriptional upregulation of genes involved in collagen synthesis and fibrosis [198,199,200]. The renin–angiotensin–aldosterone system (RAAS), in particular angiotensin II via the angiotensin type 1 (AT1) receptor, promotes fibroblast activation and TGF-β upregulation [197,200]. Moreover, angiotensin II activates NADPH oxidase, resulting in the generation of reactive oxygen species (ROS), which further amplify pro-inflammatory and profibrotic signaling cascades.

Therefore, targeting fibroblasts’ proliferation might have the potential to confer myocardial fibrosis [201]. Previous studies in HFpEF patients using anti-fibrotic medications, such as ACE inhibitors, ARBs, and aldosterone antagonists, showed some long-term benefit in reducing fibrosis but did not reduce mortality [202,203]. In 35 patients with acute myocarditis and normalized LV-EF from the initial LV-EF < 45% taking ACE inhibitors, prolonged treatment with ACE inhibitors was associated with lower incidence of new episodes of HFrEF < 45%, compared with patients who stopped taking ACE inhibitors (5% vs. 33%, *p* = 0.064), and their LV-EF was higher at 3-years follow-up (57 ± 11% vs. 47 ± 12%, *p* = 0.002), with no difference in mortality rate [202]. These results suggest that ACE inhibitors should be continued over the long term in these patients. The results of a meta-analysis of six studies, including a total of 706 patients, suggested that ACE inhibitors can effectively inhibit collagen synthesis and deposition in the myocardium, potentially preventing or even reversing the progression of myocardial fibrosis [203]. ARBs mitigate fibrosis by inhibiting the AT1 receptor and reducing cytokine expression. This blockade not only reduces afterload and blood pressure but also directly attenuates fibroblast activation and decreases TGF-β–driven collagen synthesis. Additionally, ARBs have been shown to dampen the production of pro-inflammatory cytokines, highlighting their dual hemodynamic and anti-inflammatory effects [204]. Seko et al. demonstrated an anti-inflammatory effect of the ARB olmesartan on the development of murine acute myocarditis caused by viral infection with CVB3 [204]. In the mouse model, treatment with olmesartan was associated with decreased expression of IFN-γ, iNOS, Fas ligand, and pore-forming protein, as well as lowered expression of CVB3 genomes [204]. ARNIs provide additional anti-fibrotic effects by inhibiting neprilysin, thereby increasing natriuretic peptide levels, such as BNP, which act via cGMP to inhibit fibroblast proliferation and stimulate ECM degradation [205,206].

However, the anti-fibrotic properties of ACE inhibitors, ARBs, and ARNI are moderate. One of the potentially strong experimental treatments against myocardial fibrosis involves C-type natriuretic peptide (CNP) administration [207]. Cardiac fibroblasts treated with TGF-β, with or without CNP, showed CNP-activated cGMP–PKG signaling, inhibiting TGF-β–induced myofibroblast differentiation and ECM production [207]. Tranilast, a synthetic derivative of a tryptophan metabolite, reduces cardiomyocyte injury induced by ischemia–reperfusion via Nrf2/HO-1/NF-κB signaling (Table 3) [208]. Tranilast, in a mouse model of CVB3-induced myocarditis, showed reduced myocardial fibrosis by decreasing the number of mast cells, inhibiting the expression of TGF-β1 and osteopontin, a biomarker of outcomes [209].

**Table 3:** Approaches for ARNI use and anti-fibrotic treatment in heart failure and myocarditis.

Study	Medication/Comparator	Type of the Study	Study Design	Main Findings	Outcomes	Remarks/Limitations
ARNI						
PARADIGM-HF [161]	Sac/Val vs. enalapril	RCT phase 3	In total, 8442 patients with chronic HF, NYHA class II–IV symptoms, an elevated plasma BNP or NT-proBNP level, and an LVEF of ≤35%	The primary outcome of CVD or hospitalization for HF was significantly lower in the ARNI arm compared with the enalapril arm (21.8% vs. 26.5%; HR, 0.80; 95% CI, 0.73 to 0.87; *p* < 0.001).	ARNI use reduced the risk of CVD by 16%, hospitalization for HF by 21% and decreased the symptoms and physical limitations of HF.	Study terminated earlier due to high benefits from ARNI use. Only 0.7% of patients in the NYHA functional class IV had symptoms
OUT-STEP [162]	Sac/Val vs. enalapril	Observation-al study	A total of 621 ambulatory patients with stable symptomatic HFrEF were randomized 1:1 to Sac/Val (n = 310) or enalapril (n = 311)	The study found no difference between the effect of Sac/Val vs. enalapril on 6-min walk test (6MWT) distance, non-sedentary daytime physical activity, and HF symptoms	No significant benefit of Sac/Val compared with enalapril on either 6MWT or daytime physical activity after 12 weeks	Only 0.7% of patients in the NYHA functional class IV had symptoms
PIONEER-HF trial [163]	Sac/Val vs. enalapril	RCT phase 3	A total of 736 hospitalized patients were treated for acute decompensated HF with HFrEF after stabilization.	A greater reduction in the NTproBNP, hs-TnT, and a lower rate of rehospitalization for HF in Sac/Val treatment compared to enalapril treatment was observed at 4 and 8 weeks	Fewer hospital admissions for HF in the Sac/Val arm	Patients hospitalized for acute HF after stabilization, irrespective of HF background
LIFE trial [164]	Sac/Val vs. valsartan	RCT phase 3	In total, 335 patients with HFrEF and recently advanced HF (NYHA class IV)	Compared to valsartan, treatment with Sac/Val has not improved the clinical composite of number of days alive, out of hospital, and free from HF events (*p* = 0.450).	The results showed that Sac/Val was not superior to valsartan and had a 29% discontinuation rate during the 24 weeks of the trial	Not enrolled, a predefined group of 400 patients; it was terminated prematurely due to the COVID-19 pandemic
She et al. 2021 [171]	ARNI, ACEI, and ARB groups	Propensity score of patients included in the Hospital of Xi’an Jiaotong University database	A total of 646 eligible patients with AMI were assigned to the ARNI, ACEI, and ARB groups, respectively.	Patients receiving ARNI had significantly lower rates of the composite cardiovascular outcome of CVD, MI, HF hospitalization, and IS than ACEI [HR, 0.51, 95% CI, 0.27–0.95, *p* = 0.02], and ARB users [HR 0.47, 95% CI, 0.24–0.90, *p* = 0.02]. Patients receiving ARNI showed lower rates of CVD than ACEI [HR, 0.37, 95% CI, 0.18–0.79, *p* = 0.01] and ARB users [HR, 0.41, 95% CI, 0.18–0.95, *p* = 0.04].	Subgroup analysis indicated that patients with LVEF < 40% benefit more from ARNI as compared with ACEI [HR 0.30, (95%CI, 0.11–0.86), *p* = 0.01] or ARB [HR 0.21, (95%CI, 0.04–1.1), *p* = 0.05]. Patients aged <60 years exhibited reduced composite endpoints [HR for ARNI vs. ARB: 0.11, (95%CI, 0.03–0.46), *p* = 0.002].	Not randomized, not specific for myocarditis
PARADISE-MI [172]	Sac/Val vs. ramipril	RCT phase 3	5661 post-MI patients with reduced LV-EF (≤40%) ± pulmonary congestion, randomized within 0.5–7 days post-infarction, followed for ~23 months	No significant difference in the primary endpoint (CV death, first HF hospitalization, or outpatient HF: 11.9% vs. 13.2%, *p* = 0.17)	Numerically fewer total HF events and coronary events with Sac/Val; higher incidence of hypotension (28% vs. 22%).	A study for myocardial infarction, not specifically in myocarditis
Doxorubicine-induced DCM						
Boutagy et al. 2020 [177]	Sac/Val vs. valsartan vs. placebo	Preclinical experimental study	DOX-induced cardiotoxicity in rats. The study aimed to compare the cardioprotective effect of ARNI, valsartan, vs. placebo	The treatment with ARNI caused lower LV-EF reduction compared with valsartan alone and placebo (*p* < 0.05). Cardiac fibrosis was similar in rats treated with valsartan alone, compared with Sac/Val, and significantly lower compared to DOX alone	Preservation of LV-EF in the group with ARNI. Sac/Val occurred more cardioprotective than Val in a rodent model of progressive DOX-induced cardiotoxicity. Val therapy alone only attenuated DOX-induced toxicity and fibrosis at the cellular level, whereas ARNI therapy preserved LV-EF and inhibited myocardial MMP activation.	A study on animal models
Dindas et al. 2021 [180]	Sac/Val pretreatment vs. doxorubicin	Preclinical experimental study	Four groups in mice (control; DOX only; Sac/Val only; Sac/Val pretreatment + DOX); Sac/Val given 80 mg/kg from day 1 before DOX (20 mg/kg at day 5)	Pretreatment with Sac/Val significantly attenuated DOX-induced ECG changes, oxidative stress, and inflammation, compared with DOX alone (*p* < 0.001). NT-proBNP levels were lower in the Sac/Val+DOX group compared with the DOX group, along with less caspase-3 apoptosis.	Sac/Val protects the cardiac electrophysiology, reducing biochemical and histologic markers of injury during DOX therapy.	A study on animal models
Kim et al. 2022 [181]	Low-dose ARNI + SGLT2i vs. monotherapy or placebo	Preclinical experimental study	Mouse model of doxorubicin-induced cardiotoxicity	Low-dose ARNI + SGLT2i improved survival, cardiac function, and reduced myocardial damage more than monotherapies or full-dose combo.	Additive value of low-dose ARNI + SGLT2i	A study on animal models
Myocarditis						
Nesukay et al. 2024 [187]	Sac/Val vs. enalapril	Prospective observational	Patients with acute myocarditis and HFrEF ≤ 40% treated with either enalapril (n = 48) or Sac/Val (n = 42), followed for 12 months with echocardiography and CMR	The Sac/Val group showed greater improvement in EF, myocardial strain, and functional capacity than the enalapril group	Improved cardiac function and exercise tolerance with Sac/Val	Non-randomized design and longer-term safety data are needed
Liang et al. 2022 [188]	Sac/Val vs. valsartan alone	Preclinical, experimental animal study	Mice induced with experimental autoimmune myocarditis; treated with Sac/Val or valsartan during the disease course.	Sac/Val significantly reduced myocardial inflammation, decreased Th17 cell differentiation, and lowered IL-1β and IL-6 expression	Reduced inflammatory cell infiltration, improved histology.	A study on animal models
Anti-fibrotic treatment						
Liu et al. 2024 [206]	Sac/Val vs. placebo	In vitro	In total, 30 patients were diagnosed with AMC (Autoimmune acute myocarditis)	Sac/Val alleviated myocardial inflammation while augmenting circulating CNP levels rather than BNP and ANP, accompanied by reductions in intracardial M1 macrophage infiltration and expression of inflammatory cytokines IL-1β, TNF-α, and IL-6	Sac/Val exerts a protective effect in myocarditis by increasing CNP concentration and inhibiting M1 macrophage polarization	C-type natriuretic peptide (CNP)
Wang et al. 2023 [208]	Tranilast	Experimental cell culture	An H/R model of H9c2 cardiomyocytes was established to simulate I/R-induced cardiomyocyte injury	Tranilast increased the viability of H9c2 cells, while decreasing I/R injury-induced cardiomyocyte apoptosis through reducing the expression levels of the Nrf2/HO-1/NF-κB signaling pathway	Tranilast decreased apoptosis, oxidative stress, and inflammatory response in H/R-induced H9c2 cells by activating Nrf2/HO-1/NF-κB signaling	
Huang et al. 2014 [209]	Tranilast	Experimental (mice)	Three subgroups of mice with CVB3-induced myocarditis, receiving tranilast (n = 24), placebo (n = 24), and 24 controls	The mRNA and protein expression of TGF-β1 and OPN was lower in the tranilast group than in the other groups	Tranilast reduced myocardial fibrosis by decreasing the number of mast cells and inhibiting the expression of TGF-β1 and OPN	
Levis et al. 2021 (PIROUETTE, NCT02932566) [210]	Pirfenidone vs. placebo	RCT phase 2	A total of 94 patients with stable symptomatic HFpEF (≥45%), elevated levels of natriuretic peptides, and CMR documented myocardial fibrosis (ECV ≥ 27%), randomized 1:1 to oral pirfenidone (n = 47) or placebo (n = 47) for 52 weeks	The primary outcome of the change in myocardial extracellular volume, from baseline to 52 weeks, was higher in the pirfenidone receiving group compared with the placebo (between-group difference, −1.21%; 95%CI, −2.12 to −0.31; *p* = 0.009)	In comparison to the placebo, pirfenidone significantly reduced myocardial extracellular volume	26% in the pirfenidone group and 30% in the placebo group experienced serious adverse events (nausea, rash, insomnia)

Abbreviations: 6MWT, 6-Minute Walk Test; ACE, Angiotensin-Converting Enzyme; ARB, Angiotensin Receptor Blocker; ARNI, Angiotensin Receptor-Neprilysin Inhibitor; BNP, B-Type Natriuretic Peptide; CI, confidence interval; CMR, Cardiac Magnetic Resonance; CNP, C-Type Natriuretic Peptide; CVD, cardiovascular death; DOX, doxorubicin; ECG, Electrocardiogram; HF, Heart Failure; HO-1, heme oxygenase-1; HR, hazard ratio; HFrEF, Heart Failure with Reduced Ejection Fraction; H/R, hypoxia/reoxygenation; hs-TnT, high-sensitivity Troponin T; I/R, ischemia–reperfusion; LVEF, Left Ventricular Ejection Fraction; MI, Myocardial Infarction; MMP, matrix metalloproteinases; NF-κB, Nuclear Factor kappa B; Nrf2, nuclear factor erythroid 2-related factor 2; NT-proBNP, N-Terminal Pro B-Type Natriuretic Peptide; NYHA, New York Heart Association; OPN, Osteopontin; RCT, Randomized Controlled Trial; Sac/Val, Sacubitril/valsartan; SGLT2i, Sodium-Glucose Co-Transporter 2 Inhibitors; TGF-β1, Transforming Growth Factor Beta-1.

Pirfenidone, a small oral anti-fibrotic agent that inhibits the activation of cardiac fibroblasts and the production of peptides, such as TGF-β, was investigated in preclinical studies, and then two small RCTs showed a mild, yet significant reduction in extracellular volume in patients receiving pirfenidone compared with placebo on CMR (Table 3) [210,211,212]. Pirfenidone, an oral anti-fibrotic agent without hemodynamic effect, was investigated in a phase 2 RCT in 94 patients with HF with preserved EF (>45%) and elevated levels of natriuretic peptides [210]. Authors found that among patients with myocardial fibrosis and HFpEF, administration of pirfenidone for 52 weeks reduced myocardial fibrosis on CMR [210]. However, reduction in myocardial extracellular volume was mild compared with the placebo group (−1.21%, 95% CI, −2.12 to −0.31%) [210].

### 3.9. MicroRNAs in VMC

MicroRNAs (miRNAs, miRs) are short, non-coding regulatory ribonucleic acids (RNAs) that inhibit translation by binding to target messenger RNA (mRNA) sequences [212,213,214,215]. As a result, they modulate the secretion of proteins and cytokines, significantly impacting metabolic homeostasis [216]. The expression of miRs during VMC is critically important, as miRs are implicated in viral replication, immune responses, and disease severity, and they can also serve as therapeutic targets to treat VMC [216]. Thus, they are key regulators of gene expression and play significant roles in host–virus interactions. Depending on the context, they can either promote or inhibit viral infections. Depending on the viral pathogen, a variety of miRs were described (Table 4) [217].

MicroRNAs identified in VMC in peripheral blood can reflect the degree of cardiomyocyte damage or be pathogen-specific, depending on invasion pathways and disease phase [217,218]. Regarding the first group, Goldberg et al. showed that, in children, levels of cardiac-associated miRNAs such as miR-208a, miR-208b, miR-499, and miR-21 exhibit upward or downward dynamics depending on the phase of enteroviral, adenoviral, or parvoviral B19 myocarditis [219]. Similarly to myocardial infarction, miRs: -1, -208a/b, and -499 are highly sensitive markers of cardiac necrosis [220,221,222]. In particular, miR-208b and miR-499 are located in close vicinity to myosin chains. Therefore, cardiomyocyte death leads to the release of these microRNAs into the bloodstream [94,223]. miR-21 is produced by many various cell lines, including endothelial, inflammatory, vascular smooth muscle cells, and fibroblasts [94]. Therefore, their action depends on the source cells. miR-21 downregulation could protect myocardial cells against LPS-induced apoptosis and inflammation [224,225]. Whereas, Yang et al. reported that miR-21 deficiency promoted inflammatory cytokine production and worsened cardiac function in cardiac ischemia [226]. In the late phase of VMC, miR-155, miR-135b, miR-190, miR-422a, miR-489, miR-590, miR-601, and miR-1290 were strongly induced in the hearts of patients with late viral persistence and progressive DCM [218].

In enteroviral CVB3 myocarditis, the most common cause of myocardial injury, many microRNAs promote CVB3 replication, including miR-19a/b, -22, -30a, -107, -126, -203, -590 [227]. microRNAs, involved in the inflammatory process identified in VMC, include miR-1, miR-155, miR-141-3p, miR-142-3p, miR-203, the miR-221/222 cluster, and miR-21 [228].

miR-155 and miR-148a were shown to reduce cardiac injury during the acute phase in humans by inhibiting the NF-κB pathway [229]. Results for miR-155 have been inconsistent in myocarditis, particularly regarding therapeutic interventions aimed at modulating its expression. It is important to notice that miR-155, which regulates the differentiation of macrophages, is highly upregulated and localized in heart-infiltrating macrophages and CD4^+^ T lymphocytes during CVB3-induced myocarditis [230,231]. M1 macrophages, induced by LPS and IFN-γ, typically produce copious amounts of pro-inflammatory cytokines (TNF-α, IL-12) and generate ROS. As such, M1 macrophages are associated with inflammation and tissue destruction. In Corsten et al.’s study, cardiac microRNAs were profiled in both human myocarditis and in CVB3-injected mice, comparing myocarditis-susceptible with nonsusceptible mouse strains longitudinally [230]. miR-155, miR-146b, and miR-21 were consistently and strongly upregulated during acute myocarditis in both humans and susceptible mice. Inhibition of miR-155 by a systemically delivered LNA-anti-miR attenuated cardiac infiltration by monocyte-macrophages, decreased *T lymphocyte* activation, and reduced myocardial damage during acute myocarditis in mice. Beyond the acute phase, miR-155 inhibition reduced mortality and improved cardiac function during 7 weeks of follow-up [230]. Despite this, there is currently limited evidence supporting the use of antagomirs targeting miR-155 outside of preclinical studies related to cardiac ischemia and atherosclerosis, where miR-155 is a key regulator of macrophage-driven inflammation.

Interestingly, some microRNAs appear to be specific to myocarditis and are associated with Th17 cell responses [231,232,233,234]. The Th17 cells are of particular importance as they secrete IL-17, therefore mediating myeloid cell recruitment and fibrosis and favoring DCM evolution [119]. Among Th17 cell-associated microRNAs, miR-1, miR-21, miR-146b, and miR-721 are candidates for antagomir-based therapy—with the potential to silence Th17 cells and thus suppress inflammatory pathways in VMC [231,232,233,234]. According to Liu et al., miR-146b is highly expressed in mice with CVB myocarditis [232]. Its inhibition reduced inflammatory lesions and suppressed Th17 differentiation. Inhibiting miR-146b may lead to a reduction in the severity of myocarditis [231]. A similar beneficial influence of miR-21 silencing on DCM evolution was demonstrated by Xu et al. [233]. Blanco-Domínguez et al. reported increased expression levels of miR-721 in a murine model of viral/autoimmune myocarditis compared with mice with induced myocardial infarction [234]. Notably, miR-721, synthesized by Th17 cells, was detectable uniquely in the plasma of mice with myocarditis but absent in infarcted mice [234].

Some microRNAs were identified with anti-inflammatory potential, such as miR-425 and miR-1/133a [235,236]. In CVB3-infected mice, delivery of miR-425-3p reduced levels of IL-6, IL-12, and TNF-α, resulting in inhibited myocardial inflammation and cardiomyocyte apoptosis [235]. IL-6 is fundamental for Th17 cells differentiation [119]. VMC rat models treated with miR-133a showed lower levels of inflammatory factors than non-treated groups [236]. Notably, the highest levels of these cytokines were observed in the miR-133a-silenced rats [236].

However, CVB3-induced inflammatory pathways can differ from other viral pathogens with regard to the expression levels of microRNAs (Table 4) [59,237,238,239,240,241,242,243,244]. For instance, in hMPV infection, 142 miRs were upregulated, and 32 were downregulated [238]. Notably, let-7f was significantly upregulated and exhibited antiviral effects: its inhibitors increased viral replication, whereas its mimics reduced it. The viral M2-2 protein regulated miRNAs such as miR-16 and miR-30a: miR-16 regulation depended on type I IFN signaling, whereas miR-30a was IFN independent, suggesting potential therapeutic targets [217]. Furthermore, hsa-miR-4634 enhances viral immune evasion by inhibiting type I IFN responses and interferon-stimulated genes, increasing viral replication in macrophages and epithelial cells [59]. In hMPV, the production of neutralizing antibodies depends on CD4+ T cells; therefore, this can lead to the development of new therapeutic strategies by attenuating Th17 cells and IL-17 production, thus preventing progression of VMC into DCM.

In patients with SARS-CoV-2 infection, high miR-335-3p expression level was a potential biomarker to predict disease severity [240]. In Parvovirus B19 infection, five top microRNAs associated with infection progression—miR-4799-5p, miR-5690, miR-335-3p, miR-193b-5p, and miR-6771-3p—were highly expressed in the B19V transcripts [241]. In respiratory syncytial virus (RSV), miR-146a-5p and miR-29a were upregulated, whereas let-7c, miR-345-5p, and miR-221 were downregulated by prolonged RSV infection [242,243]. miR-29a facilitated RSV replication [243].

Chen et al. investigated patients suffering from fulminant myocarditis, comparing them to healthy individuals. The increased levels of miR-29b and miR-125b in plasma were observed in the first group. Interestingly, this upregulation was positively correlated with the area of myocardial edema and was negatively correlated with the LV-EF [244]. However, miR-29b demonstrated higher sensitivity and specificity for the diagnosis of fulminant myocarditis than miR-125b [244].

Finally, a major group of microRNAs is involved in cardiac fibrosis [196]. This group is diverse, but special attention has recently focused on miR-132, a profibrotic microRNA, and currently the only one under RCT investigation as a potential target to inhibit fibroblast proliferation [245,246]. A first-in-human trial confirmed the safety and tolerability of a novel antisense oligonucleotide directed against miR-132-3p [247]. The antagomir CDR132L, currently under investigation in phase 2 RCT, demonstrated cardioprotective effects by preventing post-MI remodeling in both murine and porcine models of acute ischemia [245,246,247,248]. A phase I clinical trial further supported its therapeutic potential, showing preserved cardiac function and reversal of structural remodeling in patients with HF (Table 4) [246]. Based on these promising findings, the antagomir against miR-132-3p has entered phase 2 trials and is considered one of the few microRNA-based therapies with high potential for clinical application [248]. However, recently published data did not show any benefit from anti-miR-132 on ventricular function in humans.

In conclusion, the identification of the virus pathogen is a key determinant of immune cell and microRNA engagement. Therefore, only identification of the causal pathogen can lead to effective pathogen elimination through anti-pathogen-specific treatment.

**Table 4:** Expression of microRNAs associated with viral pathogens.

Study	Pathogen	microRNA	Down vs. Upregulated	Rationale for Use of Individual microRNA	Therapeutic Approach
Goldberg et al. 2018 [219]	enteroviral, adenoviral, or parvoviral B19 myocarditis	miR-208a, miR-208b, miR-499, and miR-21	Up or Down	upward or downward dynamics depending on the phase of infection	No data
Gong et al. 2023 [224]		miR-21	Down	miR-21 downregulation protects myocardial cells against LPS-induced apoptosis and inflammation through Rcan1 signaling	No data
Li et al. 2022 [225]		miR-21	Down	miR-21 downregulation protects myocardial cells against LPS-induced apoptosis and inflammation by targeting Bcl-2 and CDK6	No data
Yang et al. 2018 [226]		miR-21	Down	miR-21 deficiency promoted inflammatory cytokine production and worsened cardiac function in cardiac ischemia through targeting KBTBD7	No data
Bao et al. 2014 [229]	Coxsackie B3 myocarditis	miR-155, miR-148	Up	miR-155 and miR-148a were shown to reduce cardiac injury during the acute phase in humans by inhibiting the NF-κB pathway	miR-155 reduced cardiac myoblast cytokine expression. Increased survival in miR-155-treated mice
Corsten MF et al. 2012 [230]	CVB3 myocarditis in humans and susceptible mice	miR-155, miR-146b, miR-21	Up	Inhibition of miR-155 by a systemically delivered LNA-anti-miR attenuated cardiac infiltration by monocyte-macrophages, decreased T lymphocyte activation, and reduced myocardial damage during acute myocarditis in mice	LNA-anti-miR-155 may reduce inflammation activity in mice with CVB3
Zhang Y et al. 2016 [231]	CVB3 myocarditis	miR-155	Up	miR-155 is upregulated in CVB3 myocarditis, and localized primarily in heart-infiltrating macrophages and CD4+ T lymphocytes, promoting macrophage polarization to pro-inflammatory M1. Silencing miR-155 led to increased levels of alternatively activated macrophages (anti-inflammatory M2)	miR-155 may be a potential therapeutic target for VMC
Liu et al. 2013 [232]	CVB3 myocarditis	miR-146b	Up	miR-146b was highly expressed in mice with CVB3. Its inhibition reduced inflammatory lesions and suppressed Th17 cells differentiation	Inhibiting miR-146b may lead to a reduction in the severity of myocarditis
Blanco-Domínguez et al. [234]	A murine model of viral/autoimmune myocarditis in mice	miR-721	Up	Increased expression levels of miR-721 in a murine model of viral/autoimmune myocarditis. miR-721, synthesized by Th17 cells, was detectable in the plasma of mice with myocarditis but absent in infarcted mice. A murine model of viral/autoimmune myocarditis in mice	Antagomir miR-721: potential to silence Th17 cells and thus suppress inflammatory pathways in VMC
Li, J. et al. 2021 [235]	CVB3-infected mice	miR-425-3p	Up	Reduction in IL-6, IL-12, and TNF-α in VMC mice treated with miR-425-3p compared to non-treated VMC mice	miR-425-3p inhibits myocardial inflammation and cardiomyocyte apoptosis in CVB3 myocarditis
Li W et al. 2020 [236]	CVB3-infected mice	miR-1/133a	Up	miR-1/133 mimics upregulated the expression of miR-1 and miR-133, the potassium channel genes Kcnd2 and Kcnj2, as well as Bcl-2, and downregulated the expression of the potassium channel suppressor gene Irx5, L-type calcium channel subunit gene a1c (Cacna1c), Bax, and caspase-9 in the myocardium of VMC mice. miR-1/133 also upregulated the protein levels of Kv4.2 and Kir2.1, and downregulated the expression of CaV1.2	miR-1/133 mimics attenuate cardiomyocyte apoptosis and electrical remodeling in mice with VMC
Deng et al. 2014 [238]	hMPV infection	142 miRs upregulated, 32 miRs downregulated let-7f	Up Down Up	let-7f was significantly upregulated and exhibited antiviral effects: its inhibitors increased viral replication	Let-7f mimics reduced viral replication
Wu et al. 2020 [239]	hMPV infection	miR-16, miR-30a	Up	miR-16 regulation depended on type I IFN signaling, whereas miR-30a was IFN independent, suggesting potential therapeutic targets	No data
Martínez-Espinoza et al. 2023 [59]	hMPV infection	miR-4634	Up	hsa-miR-4634 enhances viral immune evasion by inhibiting type I IFN responses and IFN-stimulated genes, increasing viral replication in macrophages and epithelial cells	No data
Srivastava et al. 2023 [240]	SARS-CoV-2	miR-335-3p	Up	miR-335-3p expression level predicted COVID-19 infection severity	No data
Salvado et al. 2025 [241]	Parvovirus B19	miR-4799-5p, miR-5690, miR-335-3p, miR-193b-5p, and miR-6771-3p were highly expressed in the B19V transcripts	Up	promising biomarkers of infection progression	No data
Eilam-Frenkel et al. 2018 [242]	Respiratory syncytial virus (RSV)	miR-146a let-7c, miR-345, miR-221	Up Down	miR-146a-5p were upregulated, whereas let-7c, miR-345-5p, and miR-221 were downregulated by prolonged RSV infection	No data
Zhang Y et al. 2016 [243]	Respiratory syncytial virus (RSV)	miR-29a	Up	Respiratory syncytial virus non-structural protein 1 facilitates virus replication through miR-29a-mediated inhibition of IFN-alpha receptor	No data
Chen et al. 2022 [244]	Fulminant myocarditis	miR-29b, miR-125b	Up	miR-29b demonstrated higher sensitivity and specificity for the fulminant myocarditis. Upregulation of miR-29b and miR-125b in the plasma of patients with fulminant myocarditis positively correlated with the area of myocardial edema and was negatively correlated with the LVEF	No data
Taubel et al. 2021 [247]	HF	miR-132	Up	Randomized, phase 1b controlled trial evaluating the impact of CDR132L (antagomir-miR-132) on cardiac function over 3 months in a chronic HF model. Treatment with CDR132L significantly improved systolic and diastolic cardiac function, reduced cardiac fibrosis, and attenuated adverse remodeling	Demonstrated the therapeutic potential of targeting microRNA-132 for HF management
HF-REVERT, 2025 [248]	HF after myocardial infarction	miR-132	Up	Randomized, phase 2, controlled study in patients with HFrEE following myocardial infarction showed the safety of antagomir-132 treatment, without an effect on LV remodeling	Demonstrated safety of the treatment with antagomir-132

CVB3, coxsackievirus B3; HF, heart failure; IFN, interferon; miR, microRNA; VMC, viral myocarditis.

## 4. Conclusions and Future Perspectives

The progression from VMC to DCM has long been recognized [249]. Despite substantial advances in immunology, virology, and diagnostic modalities, effective treatments to halt or reverse this progression remain limited [250,251,252,253].

Recent insights into inflammasomes, T-cell–mediated immune responses, and the roles of cytokines such as IL-1, IL-6, IL-12, IL-17, IL-18, and TGF-β in murine models of VMC have greatly enhanced our understanding of disease pathogenesis. Pyroptotic cell death provides a novel concept to explain myocardial injury in addition to persistent viral RNA, excessive fibrosis, and immune system–mediated mechanisms. Accordingly, a therapeutic strategy that targets both the pathogen and the downstream pathological pathways appears fully justified.

The importance of optimizing myocarditis management is underscored by the recently released 2025 ESC Scientific Guidelines for the management of myocarditis [24]. The ESC expert panel highlighted that the updated guidelines could improve diagnosis, patient care, and treatment of this underdiagnosed and potentially life-threatening condition. Nonetheless, the guidelines leave certain aspects, such as timing of follow-up cardiac magnetic resonance, evaluation of specific inflammatory markers, and some treatment strategies, to the clinician’s discretion, reflecting substantial knowledge gaps and the need for individualized patient management [254,255,256,257].

Viral myocarditis remains clinically significant due to its potential progression to DCM, a process influenced by complex interactions among viral pathogens, host immune responses, and genetic predisposition. Despite advances in immunology, virology, and diagnostics, disease-modifying therapies remain limited, emphasizing the urgent need for targeted, personalized treatment approaches.

This review identifies key cellular and molecular mechanisms underlying myocarditis pathogenesis, including inflammasome activation leading to pyroptotic cell death, T-helper cell differentiation, macrophage polarization, and microRNA-mediated regulation of inflammatory pathways. These mechanisms offer multiple potential therapeutic targets, highlighting the importance of understanding immune system processes in the development and progression of myocardial injury.

Pharmacological interventions with demonstrated anti-inflammatory and anti-fibrotic effects, such as ARNI therapy, may represent a promising first-line strategy for HF secondary to myocarditis, regardless of viral etiology. Additionally, emerging therapies—including oral NLRP3 inhibitors, anti-fibrotic agents such as pirfenidone, cytokine-targeted treatments, and microRNA-based interventions—offer potential to modulate specific pathological pathways, reduce adverse cardiac remodeling, and improve cardiac function. However, most of these approaches remain in early stages of investigation, and further experimental and clinical studies are needed to establish their safety, efficacy, and long-term impact.

In conclusion, a detailed understanding of the complex interactions between viral pathogens and host genetic and immune pathways may reveal novel therapeutic targets for antiviral therapies and vaccine development. Ongoing research is essential to translate these insights into evidence-based interventions capable of preventing disease progression, reducing inflammation and fibrosis, and enhancing long-term cardiac function in patients affected by viral myocarditis. Rigorous experimental and clinical studies are necessary to validate these approaches and determine their clinical applicability.

## Figures and Tables

**Table 1:** Detailed Clinical Time Course.

Time	Course of Myocarditis: From Symptom Onset Until 6 Months Follow-Up Visit
Day 1	First symptoms: sore throat, chest pain during coughing, productive cough, chills, muscle joint pain/stiffness, fever related to acute upper respiratory tract infection
Day 5	Initial presentation: The patient presented to the emergency department with chest pain and signs of rapidly deteriorating symptoms of HF. Admission laboratory findings: PCT 1.27 ng/mL, CRP 98.7 mg/L, IL-6 19.2 pg/mL, hs-TnT 786 ng/L, NT-proBNP 6978 pg/mL, CK 343 U/L, CK-MB 20 U/L. TTE findings: reduced systolic function with LV-EF of 45% without segmental contractile abnormalities. Initial management: infection symptoms and HF treatment initiated, primarily with ACEi and diuretics, non-steroidal anti-inflammatory agents, and proton pump inhibitor
Day 6	Positive hMPV result
Day 8 and Day 9	Further decline in LV-EF to 35%, and then to 25% on TTE, change from ACEi to ARNI, decision to perform EBM. Follow-up laboratory results: PCT 0.43 ng/mL, CRP 45 mg/L, IL-6 59.7 pg/mL, hs-TnT 435 ng/L, NT-proBNP 3794 pg/mL, CK 247 U/L, CK-MB 11 U/L
Day 10	EMB performed. Post-procedurally, the patient presented with cardiogenic shock and pericardial effusion and required continuous infusion of noradrenaline and dobutamine. I.V. ARNI was discontinued.
Day 11–14	Treatment with inotropics continued. Steady reduction in pericardial effusion, and improvement in LV-EF to 45%. Reduction in inflammatory markers: PCT < 0.05 ng/mL, CRP 17 mg/L, IL-6 4.3 pg/mL, hs-TnT 39 ng/L, NT-proBNP 1876 pg/mL, CK 65 U/L, CK-MB 12 U/L
Day 14	Clinical stabilization: Complete weaning off inotropic support
Day 15	Recurrence of infection symptoms: fever, cough, dyspnoea. Laboratory findings: new increase in inflammatory markers: PCT 0.11 ng/mL, CRP 275 mg/L, IL-6 156 pg/mL, hs-TnT 115 ng/L, NT-proBNP 2731 pg/mL, CK 146 U/L, CK-MB 13 U/L. Identified co-infection with *Streptococcus pneumoniae*. Initiated azithromycin. Started prednisone as a result of EBM findings.
Day 15–19	TEE: Steady improvement in LV-EF up to 60%. Steady reduction in inflammatory parameters, as well as the improvement of the patient’s symptoms.
Day 20	Patient’s assessment on discharge: laboratory results: PCT < 0.05 ng/mL, CRP 10 mg/L, IL-6 9 pg/mL, hs-TnT 21 ng/L, NT-proBNP 3017 pg/mL, CK 15 U/L, CK-MB 9 U/L. Optimal HF therapy
3 months F-U	Follow-up visit: no resting dyspnoea, symptom in NYHA class II, ARNI titrated to intermediate dose. Laboratory findings: PCT < 0.05 ng/mL, CRP 5.0 mg/L, IL-6 1.6 pg/mL, hs-TnT 6 ng/L, NT-proBNP 1850 pg/mL, CK-MB 6 U/L
6 months F-U	Follow-up visit: no resting dyspnoea, symptom in NYHA class II, ARNI titrated to intermediate dose. Laboratory findings: PCT < 0.05 ng/mL, CRP 3.5 mg/L, IL-6 0.58 pg/mL, hs-TnT 4 ng/L, NT-proBNP 1240 pg/mL, CK-MB 3 U/L

Abbreviations: ACEi, angiotensin-converting enzyme inhibitor; ARNI, angiotensin receptor-neprilysin inhibitor; CK, creatine kinase (reference: <170 U/L); CK-MB, creatine kinase-MB (reference: <24 U/L); CRP, C-reactive protein (reference: <5.0 mg/L); HF, heart failure; IL-6, interleukin-6 (reference: <7.0 pg/mL); hMPV, human metapneumovirus; hs-TnT, high-sensitivity troponin (reference: <14 ng/L); LV-EF, left ventricular ejection fraction; NT-proBNP, N-terminal pro B-type natriuretic peptide (reference: <125 pg/mL); PCT, procalcitonin (reference: <0.5 ng/mL).

## Data Availability

The original contributions presented in this study are included in the article. Further inquiries can be directed to the corresponding author.
